# Targeting intracellular cancer proteins with tumor‐microenvironment‐responsive bispecific nanobody‐PROTACs for enhanced therapeutic efficacy

**DOI:** 10.1002/mco2.70068

**Published:** 2025-01-19

**Authors:** Changping Deng, Jiacheng Ma, Yuping Liu, Xikui Tong, Lei Wang, Jiayi Dong, Ping Shi, Meiyan Wang, Wenyun Zheng, Xingyuan Ma

**Affiliations:** ^1^ State Key Laboratory of Bioreactor Engineering East China University of Science and Technology Shanghai P. R. China; ^2^ Key Laboratory of Systems Biomedicine (Ministry of Education) Shanghai Center for Systems Biomedicine Shanghai Jiao Tong University Shanghai P. R. China; ^3^ Department of Information Engineering The Chinese University of Hong Kong Hong Kong P. R. China; ^4^ Shanghai Key Laboratory of New Drug Design School of Pharmacy East China University of Science and Technology Shanghai P. R. China; ^5^ School of Medicine Shanghai University Shanghai P. R. China

**Keywords:** nanobody, PD‐L1 and Survivin, proteolysis targeting chimeras (PROTACs), targeted degradation, tumor microenvironment

## Abstract

Proteolysis targeting chimeras (PROTACs) are pivotal in cancer therapy for their ability to degrade specific proteins. However, their non‐specificity can lead to systemic toxicity due to protein degradation in normal cells. To address this, we have integrated a nanobody into the PROTACs framework and leveraged the tumor microenvironment to enhance drug specificity. In this study, we engineered BumPeD, a novel bispecific nanobody‐targeted PROTACs‐like platform, by fusing two nanobodies with a Furin protease cleavage site (RVRR) and a degron sequence (ALAPYIP or KIGLGRQKPPKATK), enabling the tumor microenvironment to direct the degradation of intracellular proteins. We utilized KN035 and Nb4A to target PD‐L1 (programmed death ligand 1) on the cell surface and intracellular Survivin, respectively. In vitro experiments showed that BumPeD triggers Survivin degradation via the ubiquitin‐proteasome pathway, inducing tumor apoptosis and suppressing bladder tumor cell proliferation and migration. In vivo experiments further confirmed BumPeD's robust anti‐tumor efficacy, underscoring its potential as a precise protein degradation strategy for cancer therapy. Our platform provides a systematic approach to developing effective and practical protein degraders, offering a targeted theoretical basis and experimental support for the development of novel degradative drugs, as well as new directions for cancer therapy.

## Graphical abstract

We engineered a fusion protein integrating nanobodies KN035 and Nb4A, a Furin site, and a degron sequence to selectively degrade intracellular proteins. This construct targets PD‐L1 on cancer cells, internalizes, and redirects Nb4A to bind Survivin, promoting its degradation through the ubiquitin‐proteasome pathway upon Furin cleavage. Our strategy precisely targets and degrades oncogenic proteins in cancer cells using engineered proteins.

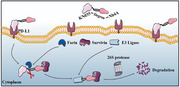

## 1 INTRODUCTION

Proteolysis targeting chimeras (PROTACs) are an innovative class of molecules that have been employed to selectively degrade proteins of interest.^1^, ^2^ In contrast to small molecule inhibitors, PROTACs can degrade any protein within the cell,^3^ including non‐druggable targets such as transcription factors and scaffold proteins.^4^, ^5^ Although promising, conventional PROTACs typically exhibit unfavorable pharmacokinetics and lack tumor specificity, which may lead to systemic toxicity due to their non‐specific distribution in normal tissues.^6^, ^7^, ^8^ Therefore, achieving targeted degradation in specific cells remains a considerable challenge.^6^, ^9^ PROTACs consist of three components, a ligand that binds to the protein of interest, a ligand that binds to the E3 ubiquitin ligase, and a linker between the two.^10^ In order to enhance the precision of conventional PROTACs and overcome their targeting limitations, it is imperative to introduce novel elements into the PROTACs framework. This strategic augmentation will facilitate the development of PROTACs capable of selectively homing in on specific tumor types.^2^, ^11^ Antibodies provide cellular targeting and can deliver drugs to target cells with greater precision, minimizing toxicity to normal tissue cells, and therefore have great promise for use in oncology and other areas.^12^ However, due to the large molecular weight of intact antibodies and their weak tissue penetration ability, there is an urgent need to introduce novel targeting protein molecules. Therefore, the introduction of novel targeting proteins based on PROTACs can offer a similar level of specificity as immunotoxin drugs. By selectively degrading their protein targets, PROTACs can achieve a precision in cellular targeting that rivals the directed action of immunotoxins.^13^, ^14^, ^15^, ^16^

Nanobodies, also known as single domain antibodies or VHH (i.e., variable domain of heavy chain) antibodies, are mainly derived from camelids and typically contain only variable regions in the heavy chain and no light chain.^17^ Compared to conventional antibodies, nanobodies offer the advantages of small size, high specificity, and stability.^18^ Capitalizing on their unique advantages, nanobodies have found extensive applications across various domains, including medical diagnostics for precise detection, therapeutics for targeted treatment, biological research for in‐depth exploration, and industrial processes for innovative solutions.^19^ They can be used to prepare highly sensitive diagnostic reagents for disease treatment, targeted drug delivery, and so forth.^20^ Hence, opting for nanobodies as a substitute for traditional antibodies not only preserves the targeting capabilities of the antibodies but also enhances their tissue penetration and stability, offering a superior alternative for various applications.

A variety of PROTACs technologies targeting nanobodies have been discovered.^21^, ^22^, ^23^, ^24^, ^25^ For example, Zhang et al.,^26^ reported a covalent nanobody‐based degradation strategy, GlueTAC (a covalent nanobody‐based PROTAC strategy), for targeted membrane protein degradation with high specificity and efficiency. Shen et al.,^21^ fused the nanobody to a cell‐permeant miniature protein, and an E3 adaptor created a degrader. However, they still cannot target specific cells. Introducing a nanobody targeting a cell surface protein could compensate for the defect. Thus, a bispecific nanobody is constructed by fusing the expression of a cell membrane protein‐targeting nanobody with an intracellular protein‐targeting nanobody. For the selection of E3 ligase ligands, the “ALAPYIP” peptide recognized von Hippel–Lindau (VHL) and could act as the ligand for VHL (named VHLL).^27^, ^28^ Also, the 14 amino acids sequence “KIGLGRQKPPKATK” (named 14aa) placed at the N‐terminal end of the protein of interest is capable of recruiting the UBR class of E3 ligases.^29^ Indeed, only when the 14aa sequence is fully exposed, the corresponding E3 ligase can be enriched, and this is done by relying on the enzymes in the tumor microenvironment to cleave the specific sequences. Furin, a highly expressed protease in tumor cells,^30^ specifically recognizes and cleaves the Arg‐X‐(Lys/Arg)‐Arg sequence after the last arginine.^31^ “RVRR” is often chosen as a substrate for Furin proteases.^32^ Thus, reengineering the constituents of PROTACs demonstrates that the incorporated nanobodies are capable of not only honing in on specific cell surface proteins but also selectively directing intracellular nanobodies to engage with and facilitate the degradation of targeted proteins. Similarly, the E3 ligase ligand serves as a crucial bridge between the two nanobodies. Furthermore, the strategic incorporation of a tumor microenvironment‐responsive cleavage peptide at the N‐terminus of the ligand leads to the development of an innovative bispecific nanobody‐targeted, tumor microenvironment‐mediated PROTACs‐like protein drug for degradation, which we term BumPeD. This approach harnesses the tumor's own microenvironment to enhance the specificity and efficacy of protein degradation.

Here, we first constructed a nanobody‐targeted PROTACs‐like platform by fusing expression of a membrane‐penetrating peptide (R_8_),^33^ Furin cleavage site (RVRR),^32^ degron sequence (VHLL^27^/14aa^29^), and a nanobody LaG16^34^ (anti‐EGFP (enhanced green fluorescent protein)) to demonstrate the feasibility of the platform using EGFP protein as an example. Subsequently, the KN035^35^ (nanobody to PD‐L1) and Nb4A^36^ (nanobody to Survivin) were selected to replace R_8_ and LaG16, respectively. Two working proteins were constructed and obtained from the BumPeD system, and a series of experiments demonstrated that these two working proteins were able to enter the cytosol through the binding of KN035 to PD‐L1, expose the VHLL or 14aa sequence through Furin protease cleavage, and then degrade the Survivin protein through the ubiquitin‐protease pathway. Overall, this platform lays the theoretical groundwork for the future development of bispecific nanobody‐targeted PROTACs‐like drugs that are activated by the tumor microenvironment. Moreover, the revelation that the degradation of the Survivin protein can prevent tumor formation offers fresh insights into considering Survivin as a promising therapeutic target. This innovative approach could pave the way for more targeted and effective cancer treatments.

## 2 RESULTS

### 2.1 Construction of the nanobody‐targeted tumor microenvironment‐mediated PROTACs‐Like system for the degradation of EGFP protein

**FIGURE 1:**
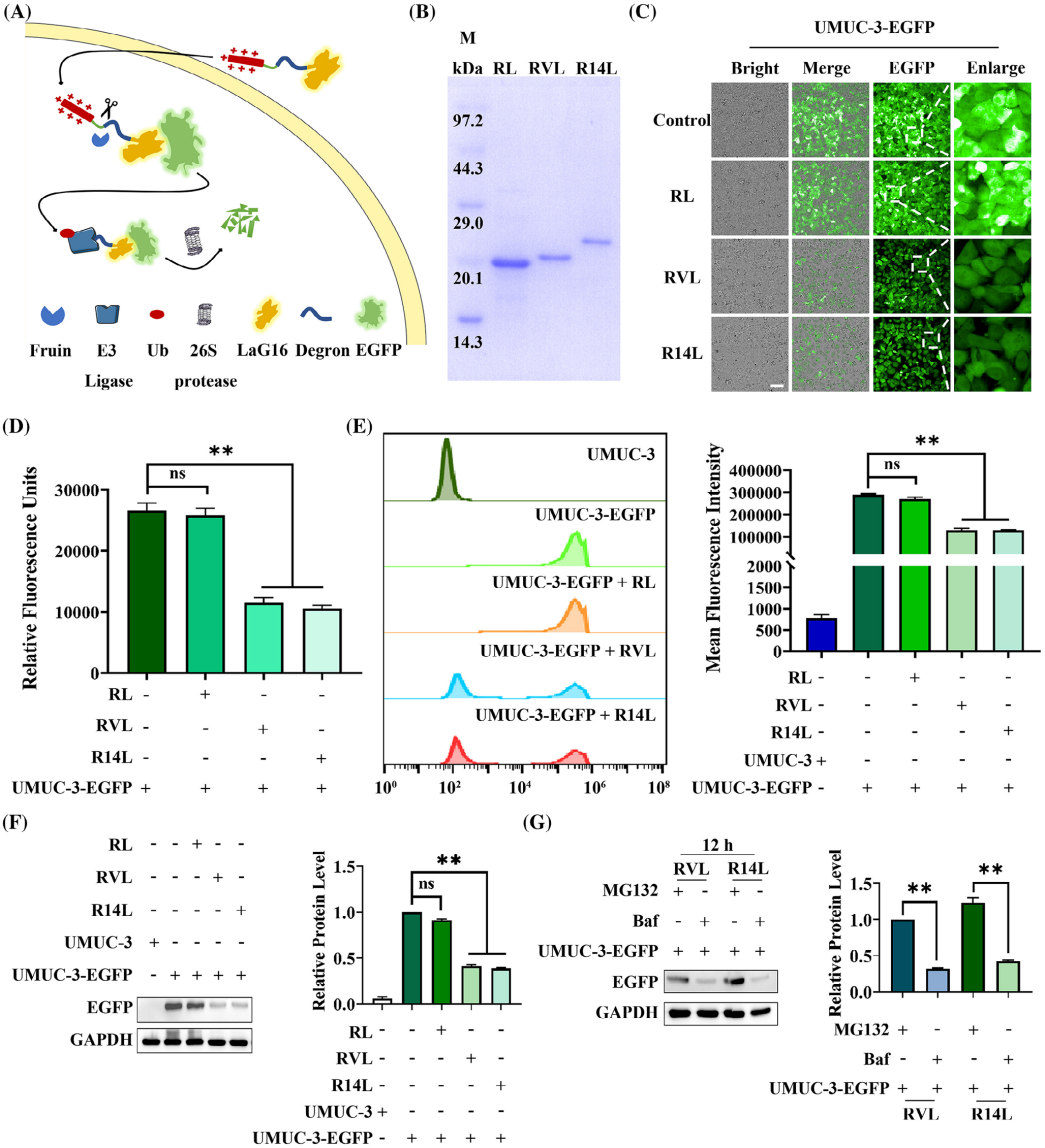
Construction and validation of the nanobody‐targeted degradation platform. (A) Nanobody‐targeted protein degradation pattern diagram. (B) Protein purification results for RL (control group), RVL (experimental group), and R14L (experimental group). (C) Fluorescence effects of RL, RVL, and R14L proteins on UMUC‐3‐EGFP cells by fluorescence inversion microscopy. The UMUC‐3‐EGFP blank group and the RL‐treated group were both control groups. (D) Effect of RL, RVL, and R14L proteins on mean fluorescence intensity of UMUC‐3‐EGFP cells by a fluorescence microplate reader. The UMUC‐3‐EGFP blank group and the RL‐treated group were both control groups. (E) Fluorescence effects of RL, RVL, and R14L proteins on UMUC‐3‐EGFP cells detected by flow cytometry. The UMUC‐3 blank group, UMUC‐3‐EGFP blank group, and RL‐treated group were control groups. (F) The effects of RL, RVL, and R14L proteins on EGFP protein expression levels in UMUC‐3‐EGFP cells were detected by western blot. The UMUC‐3 blank group, UMUC‐3‐EGFP blank group, and RL‐treated group were control groups. (G) Effects of RVL and R14L protein treatment on UMUC‐3‐EGFP cells with simultaneous exogenous addition of MG132 or Baf on intracellular EGFP protein expression levels were detected by western blot. The UMUC‐3‐EGFP cells treated with MG132 were used as controls. MG132, 10 µg/mL; Baf, 65 ng/mL. Scale bar, 100 µm. ** *p* < 0.01, ns means not significant.

Nanobodies have been selected to bind specific target proteins as ligands effectively. Thus, LaG16, a nanobody, was chosen for targeting EGFP^34^ (Figure S1). “ALAPYIP” acted as a minimal ligand for binding VHL Cullin RING E3 ligase and could efficiently enrich VHL for ubiquitin‐proteasome pathway degradation.^27^ Meanwhile, Ryan et al.,^29^ proved that the “KIGLGRQKPPKATK” amino acids sequence placed at the N‐terminal of the target protein attracts UBR1, UBR2, UBR4, and UBR5, which in turn triggered proteasome‐mediated degradation. Additionally, high expression of Furin protease in tumor cells specifically recognized the ‐RVRR‐ sequence and cleavaged after the last arginine.^32^ Furthermore, the eight positively charged arginines allowed the fusion protein to cross the cell membrane at will and enter the cells.^33^ After combining the above elements, a PROTACs‐like system that can rely on nanobody targeting was constructed (Figure 1A). To validate the pattern diagram, three expression plasmids were constructed and named RL (as a control), RVL (as an experimental group), and R14L (as an experimental group), respectively (Figure S2). Meanwhile, the three proteins were expressed, and the purity of >95% of proteins was obtained for subsequent experiments (Figure 1B).

The purified RL, RVL, and R14L were added to equal amounts of UMUC‐3‐EGFP cells at the same concentration. After 48 h of treatment, fluorescent inverted microscopy (Figure 1C), fluorescence microplate reader detection (Figure 1D), flow cytometry assay (Figure 1E), and western‐blot analysis (Figure 1F) were performed. The UMUC‐3‐EGFP blank group and the RL‐treated group were both control groups. Among them, the fluorescence units of the microplate reader exhibited that RVL and R14L reduced the fluorescence units to 43.5% and 39.8%, respectively. Meanwhile, flow cytometry analysis demonstrated that the average fluorescence intensity dropped to around 45.0%. Moreover, the western blot indicated that the expression level of EGFP protein decreased to about 40.0% under RVL and R14L treatment. The results above showed that RVL and R14L could effectively degrade EGFP protein. Subsequently, the expression levels of EGFP proteins in UMUC‐3‐EGFP cells treated with the additional addition of MG132 (proteasome inhibitor) or Baf (lysosomal and autophagy pathway inhibitor) were examined by fluorescence microscopy (Figure S3) and western blot (Figure 1G), and the results implied that RVL and R14L degraded EGFP proteins via the ubiquitin‐proteasome pathway. As a degradation platform, we can later select nanobodies to replace arginine‐penetrating peptides to target specific cells. Also, the LaG16 nanobody can be replaced to target intracellular proteins. Thus, the constructed‐BumPeD system enabled the degradation of target proteins in specific cells.

### 2.2 Validation of PD‐L1, Furin, and Survivin expression levels in multiple cells

**FIGURE 2:**
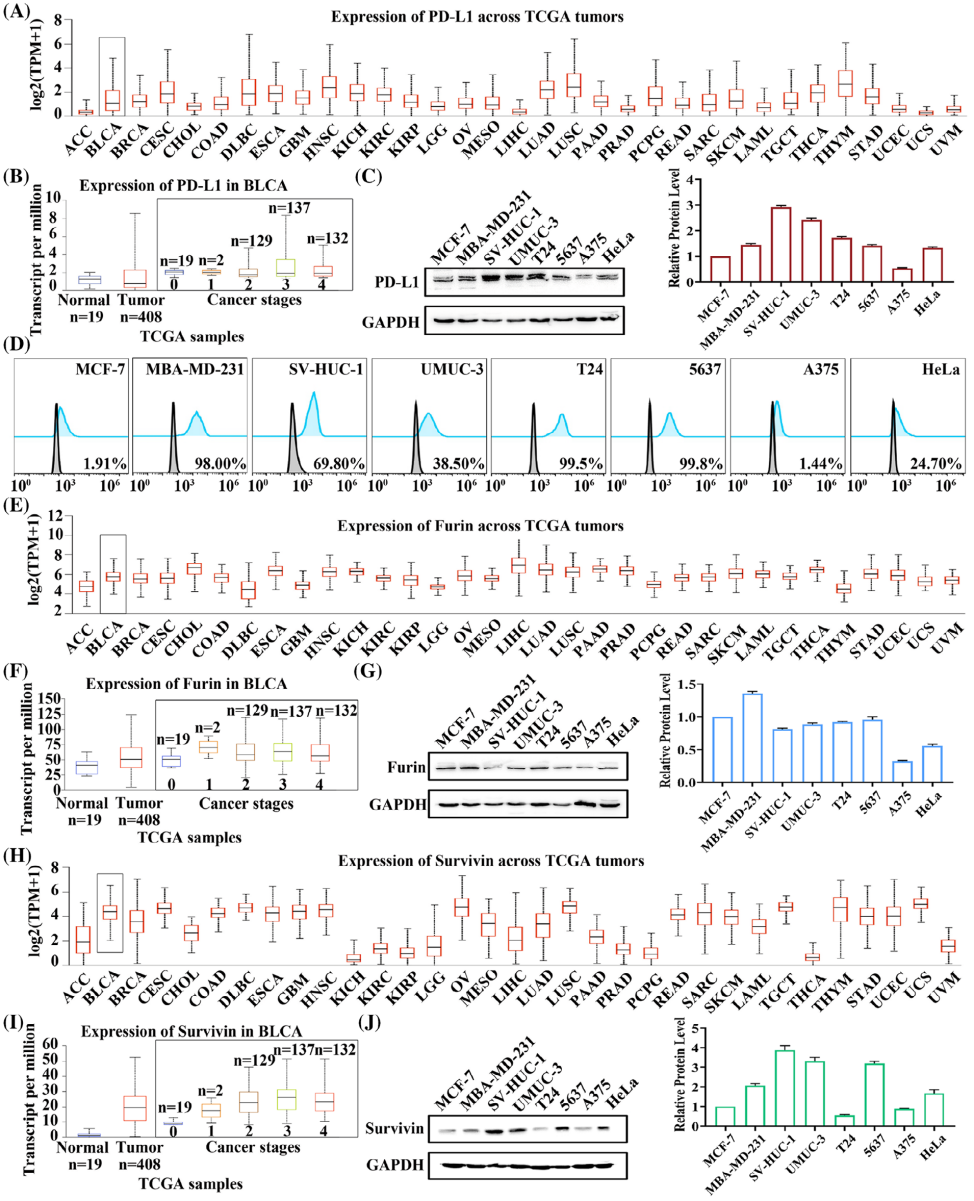
Analysis of PD‐L1, Furin, and Survivin expression levels. (A) TCGA database analysis of *PD‐L1* gene expression levels across pan cancer. (B) TCGA database analysis of *PD‐L1* gene expression levels in various stages of bladder cancer. (C) Western blot detection of PD‐L1 protein expression levels in multiple cell lines, including MCF‐7, MBA‐MD‐231, SV‐HUC‐1, UMUC‐3, T24, 5637, A375, and HeLa cells. (D) Flow cytometry measured PD‐L1 protein expression levels on the surface of multiple cell lines, including MCF‐7, MBA‐MD‐231, SV‐HUC‐1, UMUC‐3, T24, 5637, A375, and HeLa cells. (E) TCGA database analysis of *Furin* gene expression levels across pan cancer. (F) TCGA database analysis of *Furin* gene expression levels in various stages of bladder cancer. (G) Western‐blot detection of Furin protein expression levels in multiple cell lines, including MCF‐7, MBA‐MD‐231, SV‐HUC‐1, UMUC‐3, T24, 5637, A375, and HeLa cells. (H) TCGA database analysis of *Survivin* gene expression levels across pan cancer. (I) TCGA database analysis of *Survivin* gene expression levels in various stages of bladder cancer. (J) Western‐blot detection of Survivin protein expression levels in multiple cell lines, including MCF‐7, MBA‐MD‐231, SV‐HUC‐1, UMUC‐3, T24, 5637, A375, and HeLa cells.

The cell membrane protein PD‐L1 was selected as the receptor to target specific cells. It is well known that PD‐L1 is highly expressed in most cancers,^37^ which has been confirmed in the cancer genome atlas (TCGA) database (Figure 2A). Meanwhile, PD‐L1 expression was further examined in bladder urothelial carcinoma (Figure 2B), and the results showed that PD‐L1 expression levels were upregulated with increasing stages. Subsequently, MCF‐7 (as control), MBA‐MD‐231, SV‐HUC‐1, UMUC‐3, T24, 5637, A375, and HeLa cells were selected to validate the protein expression levels of PD‐L1, where SV‐HUC‐1, UMUC‐3, T24 and 5637 were bladder cell lines. Western‐blot analysis indicated that 5637 had the lowest total PD‐L1 expression level in the four bladder cell lines (Figure 2C). However, when PD‐L1 expression on the cell membrane surface was detected by flow cytometry assay, the results showed that 5637 possessed the highest expression level of 99.80%. In comparison, A375 had the lowest expression level of only 1.44% (Figure 2D). Similarly, we analyzed the expression level of Furin and showed that Furin was highly expressed in bladder cancer and was higher than controls at all stages (Figure 2E,F). Western‐blot analysis revealed that Furin expression levels were similar between 5637 and UMUC‐3 cells in the four bladder cell lines (Figure 2G). Immediately afterward, the expression levels of Survivin in pan‐cancer were analyzed. It was highly expressed in bladder cancer and upregulated with increasing stage (Figure 2H,I). Then, western‐blot results indicated that T24 was expressed at the lowest level in these four bladder cell lines, while 5637 was expressed at similar levels to UMUC‐3 cells (Figure 2J). Similarly, PD‐L1, Furin, and Survivin protein expression levels were analyzed in the Human Protein Atlas database. They showed high expression of all three proteins in bladder cancer tissues (Figure S4). Analysis of the results above revealed that 5637 could be used as a highly PD‐L1‐expressing cell, UMUC‐3 as a lowly PD‐L1‐expressing cell, and A375 as a PD‐L1 negative cell. Also, Furin and Survivin were expressed at comparable levels in UMUC‐3 and 5637 cells. Therefore, 5637, UMUC‐3, and A375 cells were selected for subsequent experiments.

### 2.3 Internalization efficiency of KN035‐targeted PD‐L1 and the distribution level of fluorescent protein in cells

**FIGURE 3:**
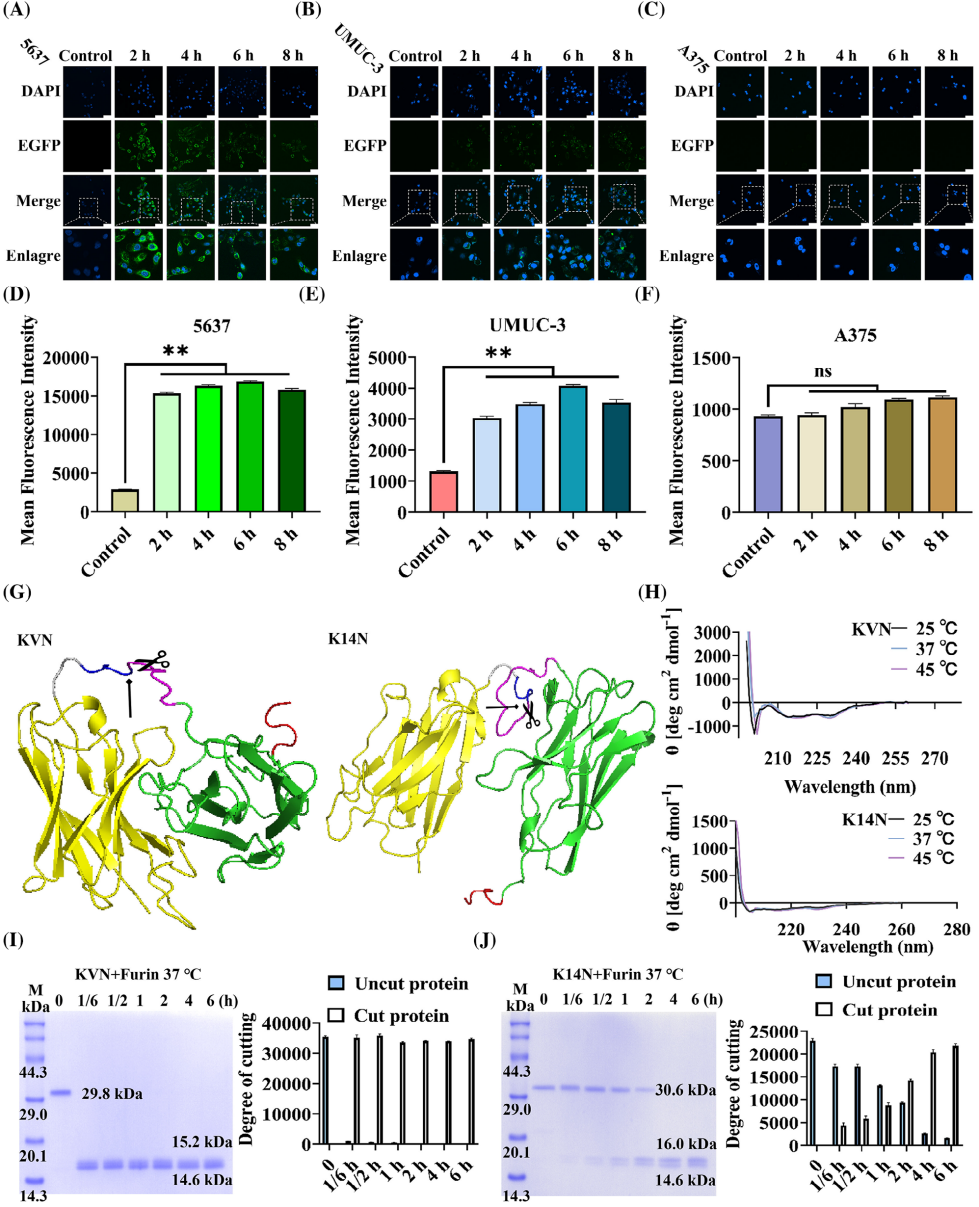
Characterization of the performance of the components of the BumPeD system. (A–C): A laser confocal scanning microscope observed the internalization levels of the KE protein on 5637 cells, UMUC‐3 cells, and A375 cells. The 0 h as a control. (D–F): Flow cytometry measured the mean fluorescence intensity of KE protein internalized in 5637 cells, UMUC‐3 cells, and A375 cells. The 0 h as a control. (G) Tertiary structure prediction of KVN and K14N proteins, with the position indicated by the arrow being the Furin protease cleavage site. (H) Structural stability of KVN and K14N proteins at different temperatures was analyzed by circular dichroism. (I) In vitro analysis of the cleavage efficiency of KVN protein by Furin protease. Furin treatment for 0 h as a control. (J) In vitro analysis of the cleavage efficiency of K14N protein by Furin protease. Furin treatment for 0 h as a control. Scale bar, 50 µm. ** *p* < 0.01, ns means not significant.

KN035,^35^ a reported nanobody targeting PD‐L1 (Figure S1), had been approved for marketing as a monospecific antibody consisting of a fused Fc segment.^38^ Thus, KN035 was selected to replace the eight arginines membrane penetrating peptide. At the same time, the degron sequence was removed, and the fluorescent protein EGFP was chosen to replace LaG16 to tracer the state of the fusion protein upon entry into the cell. The KN035‐GSG‐RVRR‐EGFP (KE) protein was constructed and expressed (Figure S2). A confocal microscope was taken by incubating KE proteins with selected cell lines at different times. As shown in Figure 3A, at 2 h, the surface of 5637 cells was covered by green fluorescence, and dotted fluorescence could also appear within the cells, compared to 0 h. With time increased, the brightness of the fluorescence on the cell membrane gradually decreased, whereas the intracellular punctate fluorescence gradually enhanced. UMUC‐3 cells showed a similar trend, but none of the overall fluorescence intensities were as bright as 5637 cells (Figure 3B). A375 cells produced no fluorescence at any time (Figure 3C). These trends were mainly due to differences in cell surface PD‐L1 expression levels. Thereafter, the fluorescence intensity of different cells at various time was also analyzed by flow cytometry (Figure 3D–F). The results unveiled that the fluorescence intensity of 5637 and UMUC‐3 cells was highest at 6 h and significantly upregulated at all other time, compared to the control group. In contrast, the fluorescence intensity of A375 showed little difference, compared to the control group. The results above indicated that KE proteins could efficiently enter PD‐L1‐expressing cells, and the punctate fluorescence also implied that the KE protein cleaved and released the EGFP protein at specific sites.

### 2.4 Characterization of working proteins and validation of the in vitro cleavage potency of Furin protease

Nb4A^36^ is a nanobody previously screened and identified in the laboratory, explicitly targeting the Survivin protein (Figure S1). Two working proteins, KN035‐GSG‐RVRR‐VHLL‐Nb4A (KVN, 29.8 KDa) and KN035‐GSG‐RVRR‐14aa‐Nb4A (K14N, 30.6 KDa; Figure S2), were constructed by replacing the EGFP protein with Nb4A and adding the different degron sequence at the N‐terminus. The homology model was constructed using SWISS‐MODEL to locate and mark the Furin protease‐specific cleavage site “RVRR” (Figure 3G). Meanwhile, the stability of the structures of KVN and K14N proteins at different temperatures was examined by circular dichroism, and it could be found that their curves were unchanged no matter at 25, 37, or 45°C, proving that they had stable secondary structures at these temperatures (Figure 3H). The exposure of degron sequences was critical, so the efficiency of Furin protease cleavage of KVN (Figure 3I) and K14N (Figure 3J) proteins was verified using in vitro experiments. A total of 6 h of treatment, with 0 h as control. Interestingly, the cleavage efficiency of the KVN protein reached 99.1% at 10 min, whereas the cleavage efficiency of K14N was only 88.8% at 4 h. In addition, it could be seen from the gel plots that the purity of both KVN and K14N proteins was greater than 95%. The only difference between these two working proteins was the degron sequences, probably due to the 14aa sequence being longer than the VHLL sequence and the folding of the protein slightly hiding the “RVRR” cleavage site.

### 2.5 KVN and K14N proteins promoted apoptosis in PD‐L1‐positive bladder cancer cells

**FIGURE 4:**
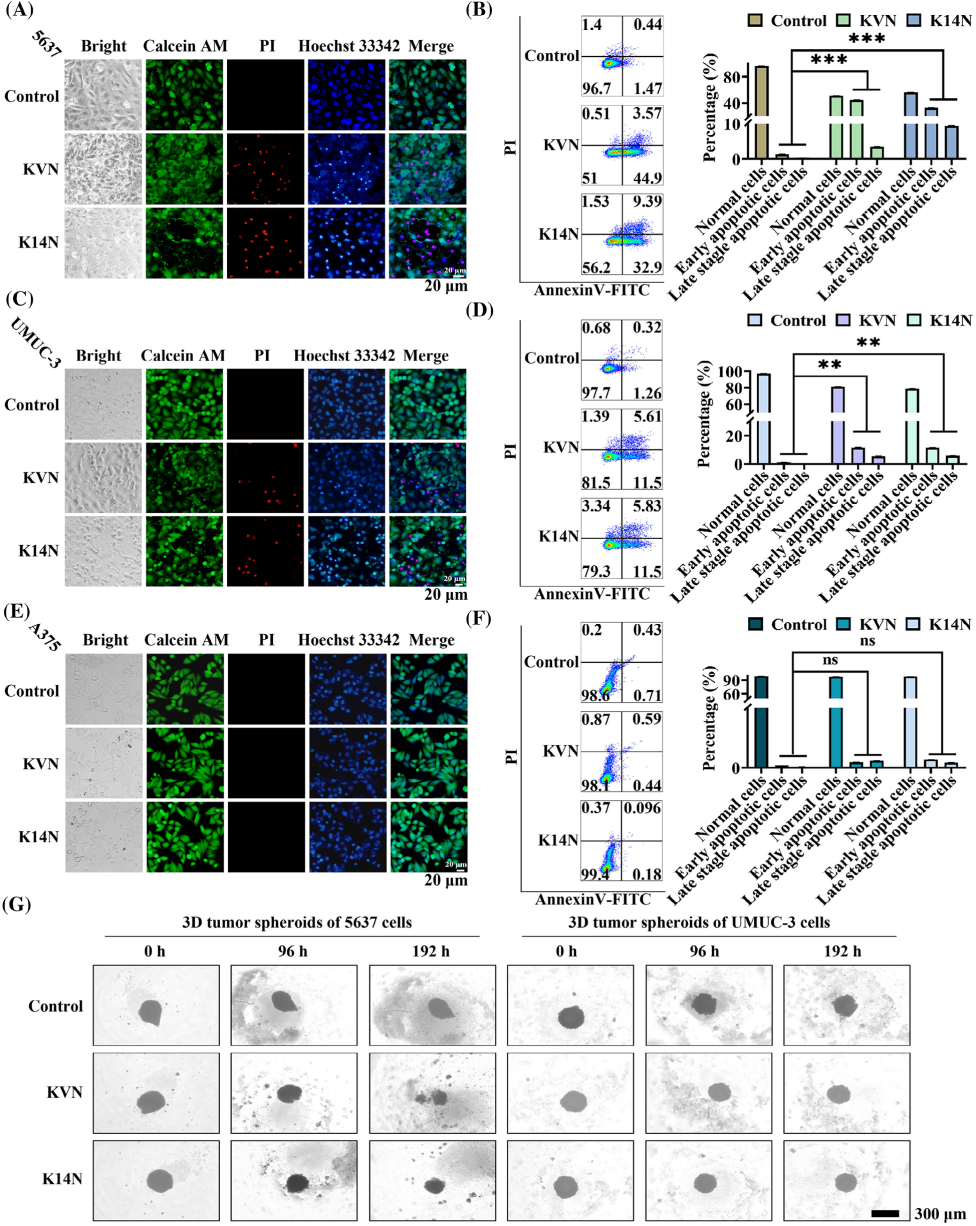
In vitro pro‐apoptotic activity assay of KVN and K14N proteins. (A, C, E) The 5637 cells, UMUC‐3 cells, and A375 cells treated with KVN and K14N were stained with Calcein/PI/Hoechst 33342 reagent, respectively. Cell lines not treated with KVN or K14N proteins were controls. Scale bar, 20 µm. (B, D, F) The 5637 cells, UMUC‐3 cells, and A375 cells treated with KVN and K14N were detected by Annexin V‐FITC/PI, respectively. Cell lines not treated with KVN or K14N proteins were controls. (G) Inhibition effect of KVN and K14N proteins on 3D tumor spheroids on 5637 and UMUC‐3 cells. Cell lines not treated with KVN or K14N proteins were controls. Scale bar, 300 µm.

Because Survivin proteins belong to the family of anti‐apoptotic proteins, degradation of Survivin proteins can promote apoptosis.^39^ Cytotoxicity experiments were performed to test this phenomenon. MTT (3‐(4,5‐dimethylthiazol‐2‐yl)‐2,5‐diphenyltetrazolium bromide) results showed that both working proteins had a killing effect on 5637 and UMUC‐3, respectively, and that the killing effect increased significantly with time and protein concentration (Figure S5A,B). However, KVN and K14N have no toxic effect on A375 cells (Figure S5C). The IC_50_ values of KVN and K14N for 5637 cells at 48 h were 84.6 and 89.8 µg/mL, respectively. 85 µg/mL was chosen as the concentration of the two working proteins for subsequent experiments. First, 5636, UMUC‐3, and A375 cells were treated with KVN and K14 for 48 h and then were stained using Calcein/PI/Hoechst 33342 and observed by fluorescence inverted microscopy. It was found that both KVN and K14N could cause apoptosis in 5637 (Figure 4A) and UMUC‐3 cells (Figure 4C), and the nucleus staining indicated that the nucleus of some cells was crumpled and chromatin was condensed. However, it did not affect A375 cells (Figure 4E). Then the three treated cells were assayed with the Annexin V‐FITC/PI apoptosis kit. Flow cytometry analysis showed that the pro‐apoptotic effects of KVN and K14N were 48.5% and 42.3% for 5637 (Figure 4B), 17.1% and 17.3% for UMUC‐3 (Figure 4D), respectively, while A375 had no pro‐apoptotic effect (Figure 4F). The results above revealed that 5637, as a PD‐L1 high‐expressing cell, could be efficiently killed by KVN and K14N, respectively, and as UMUC‐3 cells that were low‐expressed PD‐L1, KVN and K14N were still able to perform a low level of killing function. In contrast, A375, a PD‐L1 negative cell, had no pro‐apoptotic capacity with KVN and K14N treatment. Subsequently, two 3D tumor spheroid models of 5637 and UMUC‐3 cells were constructed (Figure 4G). After treating 3D tumor spheroids with KVN and K14N proteins for a certain time and observing the changes of 3D tumor spheres, it was found that both KVN and K14N proteins could effectively inhibit the volume of tumor spheres. KVN had the most significant inhibitory effect on 5637 cell tumor spheres, causing the spheroid part to lyse. The inhibitory effect of KVN and K14N proteins on the 5637 tumor spheroid was significantly more robust than that of the UMUC‐3 cell tumor spheroid, mainly due to the difference in surface PD‐L1 expression levels.

### 2.6 KVN and K14N inhibited the proliferation and migration of PD‐L1‐expressing cells, respectively

**FIGURE 5:**
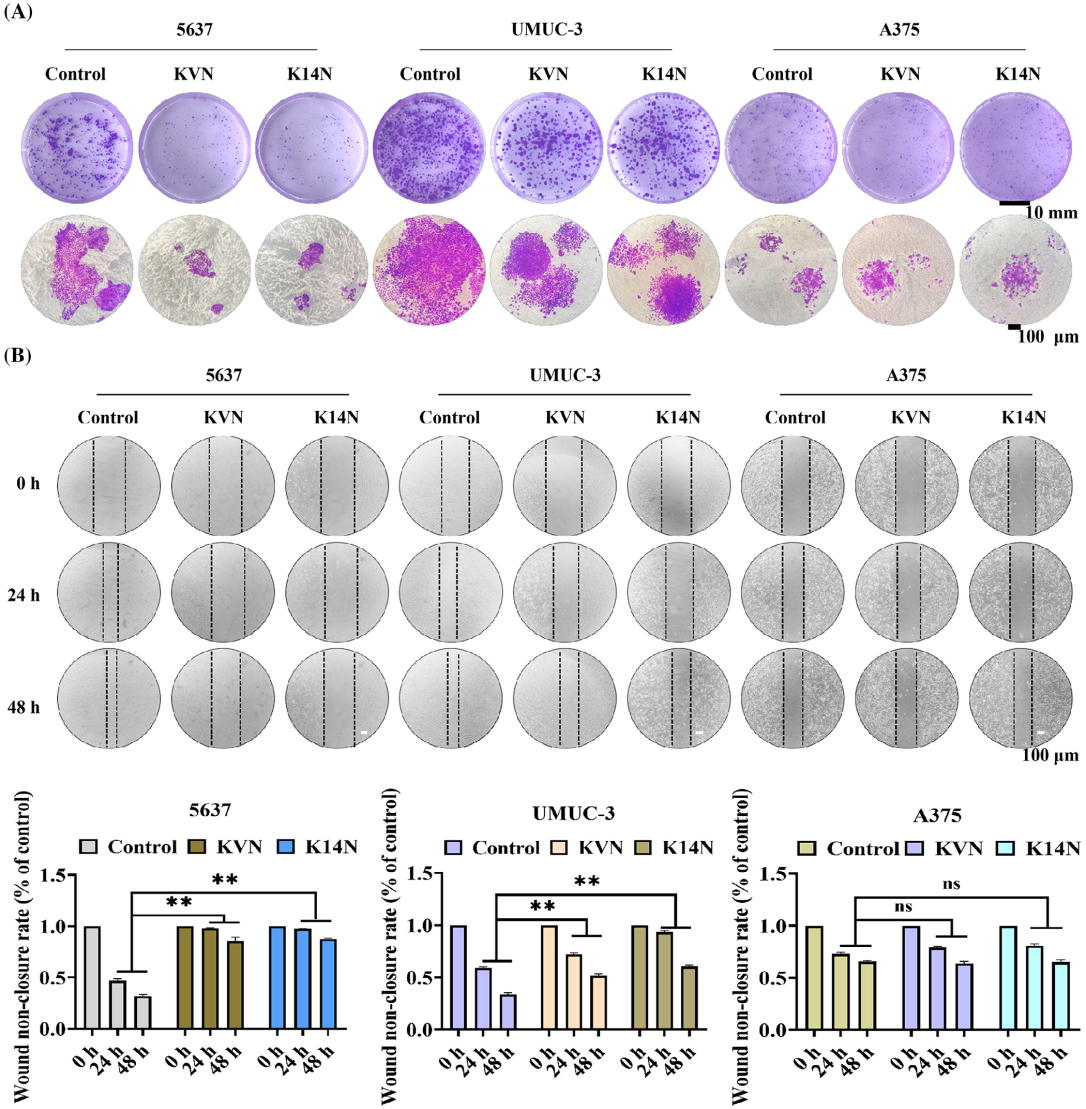
KVN and K14N proteins significantly inhibited the proliferation and migration of bladder cancer cells. (A) The effects of KVN and K14N proteins on the proliferation of 5637 cells, UMUC‐3 cells, and A375 cells were analyzed using clone formation assays with crystal violet staining. Cell lines not treated with KVN or K14N proteins served as controls. Scale bar, 10 mm, and 100 µm (B) The migratory effects of KVN and K14N proteins on 5637 cells, UMUC‐3 cells, and A375 cells were analyzed using scratch assay. Cell lines not treated with KVN or K14N proteins served as controls. Scale bar, 100 µm. ** *p* < 0.01, ns means not significant.

Survivin protein not only functions as an anti‐apoptotic agent but also promotes the proliferation and migration of cancer cells.^40^ Degradation of the Survivin protein inhibits the proliferation and migration of cancer cells. Both KVN and K14N were effective in inhibiting the proliferation of 5637 and UMUC‐3 cells by clonogenic assays. Crystalline violet staining showed that the clonal clusters of KVN and K14N treated 5637 and UMUC‐3 cells were significantly smaller, especially 5637 cells. In contrast, there was essentially no difference in the clonal clusters of A375 cells (Figure 5A). The scratch assay found that treatment of 5637 and UMUC‐3 cells with KVN and K14N resulted in varying degrees of inhibition of their scratching. The highest inhibition rate was observed in 5637 cells, followed by UMUC‐3 cells, and no inhibition in A375 cells (Figure 5B). The inhibition effect was mainly due to the difference in KVN and K14N, two working proteins entering the target cells. With the same concentration of the working protein, only the high level of PD‐L1 expression on the cell surface could make the working proteins enter intracellularly in large amounts and then exercise the subsequent functions.

### 2.7 KVN and K14N entered 5637 cells to degrade Survivin protein via the ubiquitin‐proteasome pathway

**FIGURE 6:**
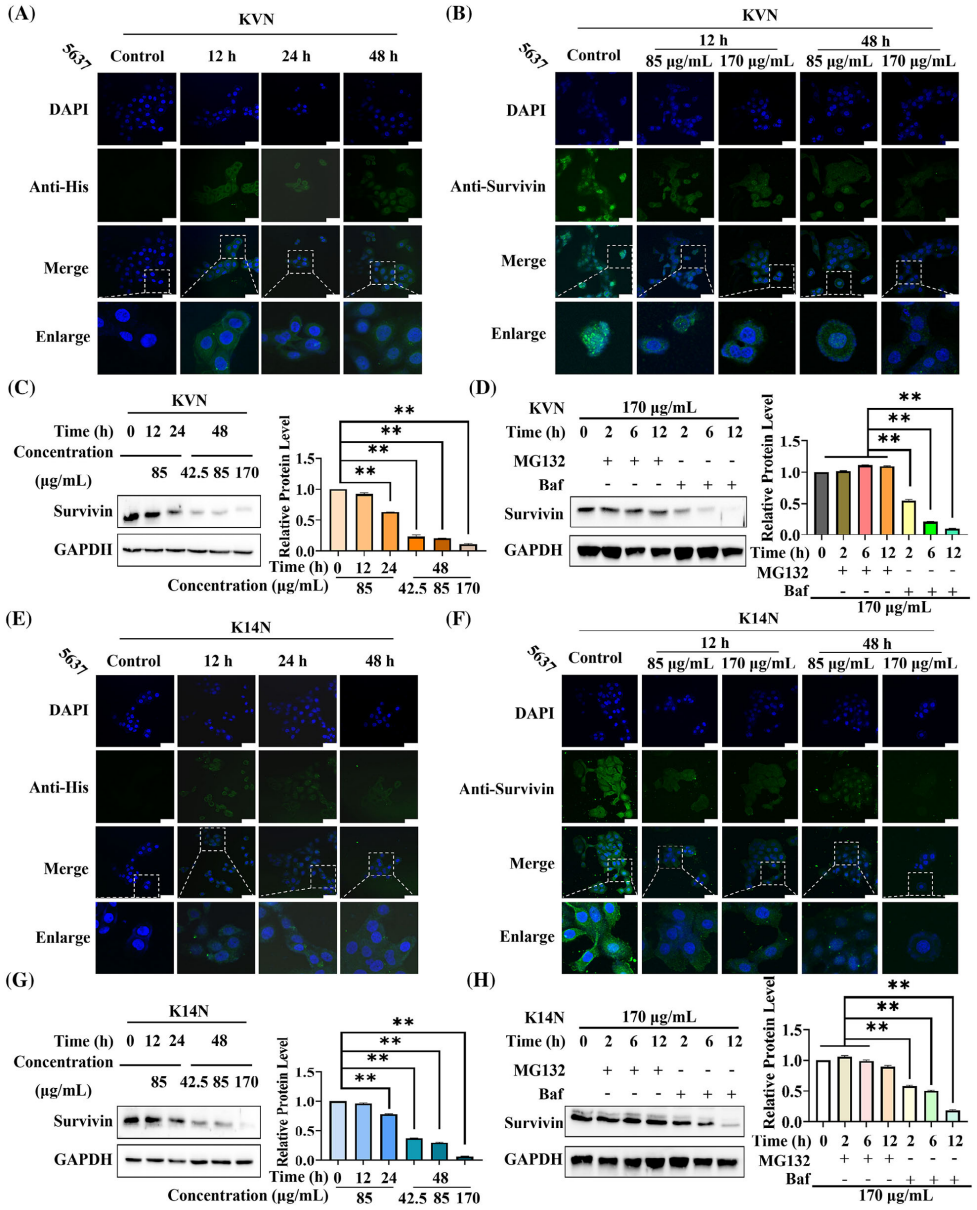
KVN and K14N proteins degraded Survivin protein via the ubiquitin‐proteasome pathway in 5637 cells. (A, E) Indirect immunofluorescence analysis of KVN and K14N proteins entry into 5637 cells. KVN or K14N protein treatment for 0 h as control. (B, F) Indirect immunofluorescence detected the effect of different concentrations of KVN and K14N proteins on Survivin protein at different times in 5637 cells. KVN or K14N protein treatment for 0 h as control. (C, G): Western blot detected the effect of different concentrations of KVN and K14N proteins on Survivin protein expression at different times in 5637 cells. KVN or K14N protein treatment for 0 h as control. (D, H): Western blot detected the effect of KVN and K14N proteins treatment of 5637 cells with simultaneous exogenous addition of MG132 or Baf on intracellular Survivin protein expression levels. KVN or K14N protein treatment for 0 h as control. MG132, 10 µg/mL; Baf, 65 ng/mL. Scale bar, 50 µm. ** *p* < 0.01.

The 5637, as a PD‐L1‐highly expressing bladder cancer cell, treating KVN with K14N proteins promoted the apoptosis of 5637 cells and inhibited the proliferation and migration. Therefore, 5637 cells were selected to verify the degradation of the Survivin protein. The working proteins with His tag were incubated with anti‐His primary antibody, with fluorescently labeled secondary antibody to investigate the state of KVN and K14N proteins after entering the cells and observed by confocal microscopy after the samples were processed. Different time points were selected for the experiment, where 0 h was the control. It could be found that the proteins containing Nb4A cleaved by Furin protease were gradually enriched intracellularly with increasing time, but they could not enter the nucleus (Figure 6A,E). Meanwhile, different concentrations of KVN and K14N were used to treat 5637 cells, and the cells were treated at different time for confocal photography. Here, the primary antibody against Survivin protein, with fluorescently labeled secondary antibody, was chosen to explore the degradation of the working proteins into the cells against the target protein. The results emerged that the expression level of Survivin protein gradually decreased with time under the treatment of KVN or K14N, which showed a gradual decrease of fluorescence intensity in the cytoplasm. Increasing the concentration of KVN or K14N simultaneously also decreased fluorescence intensity in the cytoplasm (Figure 6B,F). The western‐blot results also showed the same trend, which indicated that the degradation level of Survivin protein was positively correlated with the treatment time and treatment concentration of KVN or K14N. When 5637 cells were treated with 170 µg/mL of KVN or K14N for 48 h, the Survivin protein expression level decreased to 10.9% in the KVN‐treated group and 6.4% in the K14N‐treated group (Figure 6C,G). Meanwhile, the half degradation concentration (DC_50_) curves were plotted by western‐blot data (Figure S6), and the DC_50_ values of KVN and K14N proteins against Survivin protein in 5637 cells were found to be 5.5 µg/mL and 29.0 µg/mL, respectively. It is well known that the major degradation pathways of eukaryotic proteins include the ubiquitin‐proteasome system pathway and the lysosomal‐autophagic pathway.^41^ To investigate which pathway KVN and K14N degrade Survivin protein, 5637 cells were co‐treated with MG132^42^ or bafilomycin A1.^43^ By adding MG132 or Baf at a final concentration of 10 µg/mL and 65 ng/mL, respectively, the results showed that the expression of the target protein Survivin was essentially indistinguishable when MG132 was added and decreased with time when Baf was added, regardless of whether the cells were treated with KVN or K14N (Figure 6D,H). The results above exhibited that KVN or K14N proteins could degrade the target protein Survivin through the ubiquitin‐proteasome pathway after entering the cells. The degradation level was closely related to the treatment time with the concentration of the working proteins.

### 2.8 Evaluation of in vivo antitumor activity of KVN and K14N proteins

**FIGURE 7:**
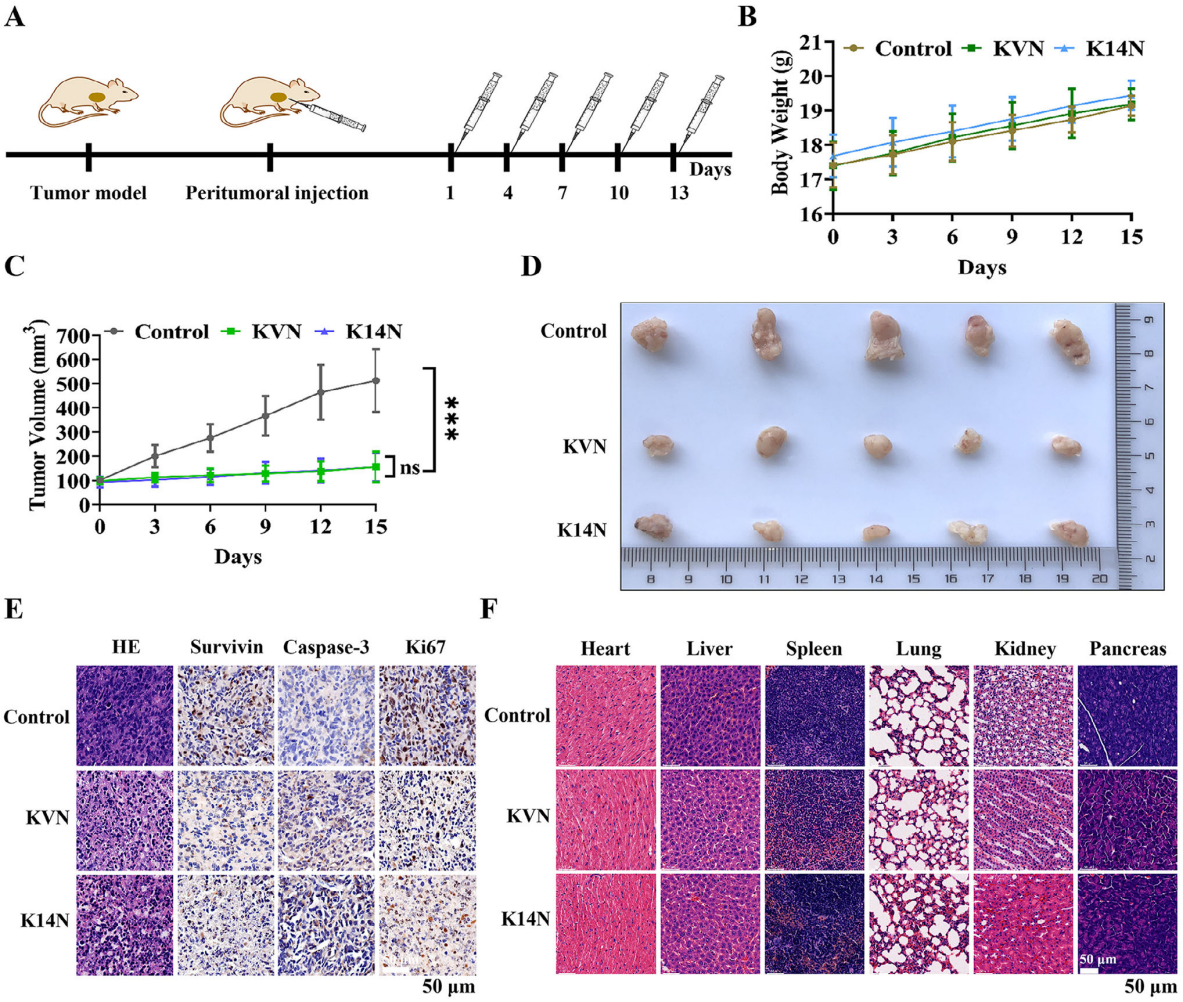
In vivo assessment of tumor inhibition by KVN and K14N proteins. (A) Overall design of animal experiments. (B) Weighing nude mice in the control and experimental groups during treatment. (C) Tumor volumes were measured in control and experimental nude mice during treatment. (D) Tumors from control and experimental nude mice were peeled and photographed 15 days after treatment. (E) HE and IHC analysis of tumors in the control and experimental groups. (F) HE staining of the principal organs of nude mice in the control and experimental groups, including heart, liver, spleen, lung, kidney, and pancreas. The control group was treated with PBS (Phosphate Buffered Saline), and the experimental group was treated with KVN or K14N protein.

A series of in vitro cellular experiments has demonstrated that KVN and K14N proteins could effectively enter 5637 and UMUC‐3 cells, and degrade Survivin protein via the ubiquitin‐proteasome pathway, thereby promoting apoptosis and inhibiting proliferation and migration. The 5637 cell line should be selected for nude mouse tumorigenesis from the effect of cell experiments. However, after many attempts, the tumor still failed to form. Given the difficulty of tumorization of 5637 cells, the UMUC‐3 cell line was often reported in the literature^44^, ^45^ and was selected for nude mouse tumorigenesis experiments. As a result, the cell‐derived xenograft (CDX) model was constructed for animal experiments. The overall experimental plan is shown in Figure 7A. When the tumor volume grew to about 100 mm^3^, KVN and K14N protein were administered, and the administration method was peritumoral administration, a total of five times with an interval of 3 days, and the nude mouse was weighed. The tumor volume was measured before the administration. Body weight measurements revealed a slow increase in the body weight of these mice, indicating that KVN and K14N proteins were less toxic and did not affect the growth of the mouse (Figure 7B). Measurement of tumor volumes showed that either the KVN or K14N protein significantly inhibited tumor growth, and there was no difference between the KVN‐treated and K14N‐treated groups (Figure 7C). Subsequently, at the end of the treatment, all of the nude mice were euthanized. Tumor blocks were removed and photographed, and it was found that both the KVN‐treated group and the K14N‐treated group were able to significantly inhibit the growth of the tumor blocks, compared to the control group (Figure 7D). Meanwhile, the results of HE (hematoxylin‐eosin) and IHC (immunohistochemistry) on the tumor blocks of the experimental and control groups demonstrated that KVN and K14N proteins were effective in reducing Survivin protein expression, similarly, that inhibition of Survivin led to the accumulation of Caspase‐3, which in turn promoted apoptosis. Down‐regulation of Ki‐67 protein implied reduced tumor proliferation capacity (Figure 7E). Also, the HE staining showed that the heart, liver, spleen, lung, kidney, and pancreas did not exhibit significant histomorphology changes in the experimental group, compared to the control group (Figure 7F). As a result, animal studies indicated that KVN and K14N proteins could reduce Survivin protein and inhibit tumor growth.

## 3 DISCUSSION

### 3.1 A novel PROTACs‐like platform could be used effectively for target protein degradation

Antibodies play a massive role in the treatment and diagnosis of cancer, and several have become first‐line therapeutic agents for certain tumors.^12^ Nanobodies are unique antibodies derived from camels, with only heavy chains, and are gradually used for chimeric antigen receptors and targeted drug delivery.^20^ Nanobodies contain many advantages, such as small size (∼15 kDa), good stability, high tissue penetration, reasonable specificity and affinity, and so forth.^17^, ^18^ Given these advantages, we fused them with conventional PROTACs to obtain a PROTACs‐like degradation system that used membrane‐penetrating peptides to enter cells, used the tumor microenvironment to mediate exposure of degron sequences, and used nanobodies to target intracellular proteins. The incorporation of nanobodies significantly bolsters the degradant's targeting capabilities, enabling it to hone in on the degradation of pivotal proteins within a specific cell type. This refined approach contrasts with conventional PROTACs, which indiscriminately mediate the degradation of certain proteins across all cells, lacking the cell‐type specificity that nanoantibody‐enhanced degradants provide. Thus, incorporating this system into UMUC‐3‐EGFP cells caused the degradation of EGFP protein. Previously, Ibrahim et al.^24^ fused the RING domain of ubiquitin E3 ligase RNF4 with a nanobody to obtain an antibody RING‐mediated destruction (ARMeD) degradation platform. This system expressly induced YFP‐PARG protein (yellow fluorescent protein‐poly ADP ribose glycohydrolase) degradation under doxycycline (DOX) induction and the fluorescence signal was substantially reduced. Also, as a nanobody‐targeted degradation system, ARMeD was transfected into cells in the form of nucleic acids and was induced by DOX to express effector proteins, which in turn were targeted to the YFP‐PARG protein via the nanobody, thereby degrading the target protein via the ubiquitin‐proteasome pathway. However, the degradation platform established in this study was in the form of a protein that directly controlled the degradation of the target protein and introduced the concept of tumor microenvironment‐mediated site‐specific cleavage, allowing the protein to spontaneously exert the degradation of the target protein in the ubiquitin‐proteasome system within the tumor cell. A comparison shows that although the degradation efficiency of this platform is lower than that of ARMeD, the concept of degradation within the tumor‐specific environment is superior to it. On this basis, we replaced the membrane‐penetrating peptide with another specific nanobody, thus constructing a degradation system called BumPeD. Because we can collectively refer to the binding of nanobodies to antigen, the binding of E3 ligase to degron ligand, and the recognition and cleavage of “RVRR” sequences by Furin protease as bumped.

### 3.2 Bispecific nanobodies implanted into PROTACs‐like system targeted specific cells and degraded intracellular proteins

To date, most PROTACs have been developed using ligands that recruit E3 ligases widely expressed in tumors and normal tissues, and these PROTACs can lead to off‐target toxicity if the protein of interest is not tumor specific.^7^ However, novel tumor‐specific E3 ligases are challenging to excavate, which requires introducing targetable elements to the existing base.^46^ For example, Marei et al.^13^ fused the concept of bispecific antibodies with PROTACs to obtain a bispecific antibody that targeted both the cell membrane surface E3 ligase RNF43 or ZNRF3 and the IGF1R protein, termed PROTABs. Zhang et al.^26^ fused the nanobodies KN035, nine arginines, and a lysosomal sorting sequence to construct the GlueTAC degradation system, which could target PD‐L1 high‐expressing cells and bring PD‐L1 into the lysosome to induce lysosomal pathway degradation. Therefore, based on the advantages of the above studies, we designed the BumPeD system for the targeted degradation of Survivin protein in PD‐L1 high‐expressing cells. This system also introduced the KN035 nanobody as a “sight” to target PD‐L1 high‐expressing cells and the Nb4A nanobody as a “bullet” to target the intracellular Survivin protein. The highly expressed Furin protease in the tumor could efficiently cleave the “RVRR” sequence to expose the degron sequence, a choice based on the tumor microenvironment.^47^ By comparing PROTABs, GlueTACs, and BumPeD, it can be found that BumPeD combines the advantages of PROTABs and GlueTACs, not only by combining bispecific nanobodies with PROTACs but also by introducing the concept of the tumor microenvironment to enhance the specificity of the target of degradation.

### 3.3 Targeted degradation of Survivin protein in PD‐L1‐expressing cells inhibited tumor growth

Survivin, a potentially effective target for cancer therapy, has been proven to promote apoptosis by inhibiting its expression^39^ and also plays an essential role in the chemotherapy resistance process.^48^ For example, Hu et al.^49^ inhibited Survivin expression at the transcriptional and protein levels to suppress tumor growth in tumor‐bearing mice. Qi et al.^40^ used CRISPR/Cas9 to edit the *Survivin* gene in vivo to treat orthotopic hepatocellular carcinoma. Also, Deng et al.^48^ indicated that Survivin might mediate the development of chemoresistance by regulating the expression of P‐glycoprotein through PI3K/Akt/mTOR (Phosphatidylinositol 3‐Kinase/Protein Kinase B/Mammalian Target of Rapamycin). In this study, establishing the BumPeD system was validated in 5637 and UMUC‐3 cells. Flow cytometry demonstrated that 5637 and UMUC‐3 cells expressed PD‐L1 protein on the surface, and 5637 cells highly expressed PD‐L1 on the surface, up to 99.8%. In addition, Furin protease and Survivin protein were abnormally highly expressed in 5637 and UMUC‐3 cells. Therefore, 5637 and UMUC‐3 cells were selected for treatment. Due to differences in cell surface PD‐L1 expression levels, KVN and K14N proteins had the most significant effect on 5637 cells. The working proteins were incubated with 5637 cells, and indirect immunofluorescence and western‐blot results showed that KVN and K14N could effectively degrade the Survivin protein in the cytoplasm. The degradation of intracellular Survivin protein promoted the apoptosis of 5637 cells and inhibited proliferation and migration. Given the difficulty of tumorigenesis of the 5637 cell line, the UMUC‐3 cell line was selected for the construction of the nude mouse CDX model. The results of animal studies demonstrated that the degradation of the Survivin protein effectively inhibited tumor growth. The BumPeD system was a flexible and variable degradation system whose components could be replaced with more practical elements, such as screening for nanobodies with greater affinity and specificity to replace existing nanobodies,^50^ which could enhance the diversity of cell membrane surface and intracellular antigens. Also, replacing degron sequences enriched with more E3 ligases to achieve more significant degradation. Finally, the replacement of short peptide sequences that could be cleaved by the tumor microenvironment with higher cleavage efficiency. Through continuous experimentation on this platform, solid and effective targeted degradation proteins can be obtained, providing a specific theoretical basis and support for developing novel degradation drugs. Nevertheless, the BumPeD platform faces certain constraints, primarily due to the nanobody screening process. Optimal therapeutic outcomes within the BumPeD system are only achievable upon the successful identification of nanobodies that possess high affinity and stability. In a similar vein, the capacity of degron sequences to recruit E3 ligases is of paramount importance; they must be able to rapidly and abundantly attract E3 ligases to effectively initiate the ubiquitin‐mediated degradation of target proteins.

## 4 MATERIALS AND METHODS

### 4.1 Cell lines and cell culture

In this study, MCF‐7, MBA‐MD‐231, SV‐HUC‐1, UMUC‐3, T24, 5637, A375, and HeLa cells were used, all purchased from the American Type Culture Collection, while UMUC‐3‐EGFP (EGFP stable transfer line) was constructed and characterized by the laboratory.^51^ All cell lines were grown in a medium containing 10% fetal bovine serum (Invitrogen) and 1% antibiotics (penicillin/streptomycin/amphotericin B; Solarbio) and placed in an incubator at 37°C with 5% CO_2_.

### 4.2 Reagents and kits

DMEM and 1640 medium for cell culture were purchased from Beijing Solarbio Science & Technology Co. The homologous recombinant kit for vector construction was purchased from TransGen Biotech Co. Quantification of protein concentration using the bicinchoninic acid protein assay kit (Sangon Biotech). The primary antibodies used in western blot were anti‐EGPF (mouse/IgG1; 1:2000; Leading Biology), anti‐GAPDH (mouse/IgG2b (Immunoglobulin G2b); 1:50,000; Proteintech), anti‐PD‐L1 (Rabbit/IgG; 1:200; ABclonal), anti‐Furin (rabbit/IgG; 1:1,000; ABclonal), anti‐Survivin (mouse/IgG1; 1:3,000; Proteintech), and the secondary antibodies used were HRP (Horseradish Peroxidase)‐conjugated affinipure goat anti‐rabbit IgG(H+L) (1:10,000; Proteintech) and HRP‐conjugated affinipure goat anti‐mouse IgG(H+L) (1:10,000; Proteintech). The polyvinylidene difluoride membranes (pore size: 0.22 µm) were purchased from Beyotime Biotechnology Co. The results of the western blot were developed on film using the high sensitive plus ECL (enhanced chemiluminescence) reagent purchased from Sangon Biotech. The antibody used in the flow cytometry assay was recombinant anti‐PD‐L1 antibody (PE), rabbit monoclonal (10 µL/test, 0.1 mg/mL, SinoBiological). Furin protease purchased from NEW ENGLAND BioLabs. Calcein/PI Cell Viability/Cytotoxicity assay kit and Hoechst 33342 were purchased from Beyotime Biotechnology Co. Annexin V‐FITC/PI apoptosis detection kit was purchased from Vazyme. The primary antibodies used for indirect immunofluorescence were anti‐His (mouse/IgG1, 1:400; Proteintech) and anti‐Survivin (mouse/IgG1; 1:200; Proteintech). Also, the secondary antibody used was goat anti‐mouse IgG(H+L) (Alexa Fluor 488; 1:200, Abcam). The PrimeSurface low adsorption cell culture plates were purchased from Whatman.

### 4.3 Methods

The complete methods of bioinformatics analysis, vector construction and protein expression, western blot, EGFP fluorescence intensity assay, protein internalisation assay, characterization of the working proteins, Furin protease cleavage analysis in vitro, MTT assay, cell apoptosis assay, cloning formation assay, cell migration assay, cell viability assays, indirect immunofluorescence assay, 3D tumor spheroid growth inhibition, and in vivo antitumor efficiency can be found in the Supporting Information.

### 4.4 Statistical analyses

Data were depicted as mean values ± standard deviation. The assessment of statistical significance was conducted utilizing one‐way ANOVA (Analysis of Variance). These analyses were performed using GraphPad Prism version 8.0 (GraphPad Software). A threshold of *p* < 0.05 was applied to define statistical significance, denoted as follows: * for *p* < 0.05, ** for *p* < 0.01, *** for *p* < 0.001, and ns indicate not significant.

## AUTHOR CONTRIBUTIONS

X.M., W.Z., and C.D. designed the study and conceived the project. C.D., Y.L., and L.W. performed the functional experiments and conducted formal data analysis. C.D., X.T., and J.D. designed and performed cell imaging experiments. C.D. collected the samples and analyzed the data. C.D., W.Z., and X.M. wrote the manuscript. J.M., P.S., and M.W. polished the manuscript. All authors have read and approved the final manuscript.

## CONFLICT OF INTEREST STATEMENT

The authors declare no conflict of interests.

## ETHICS STATEMENT

All animal experiments were approved by Chinese legislation on the use and care of research animals (Document No. 55, 2001) and controlled by the animal ethics committee of East China University of Science and Technology (REC No. 20181223). At the end of the experiments, all mice were euthanized.

## Supporting information



Supporting Information

## Data Availability

All data needed to evaluate the conclusions in the paper are present in the paper and/or the Supporting Information. Additional data related to this paper may be requested from the authors. The datasets presented in this study can be found in online repositories.
