# Supplementary material for: Targeting intracellular cancer proteins with tumor‐microenvironment‐responsive bispecific nanobody‐PROTACs for enhanced therapeutic efficacy
Source: MedComm (2020). 2025 Jan 19;6(2):e70068. doi: 10.1002/mco2.70068 (PMC11742431; doi:10.1002/mco2.70068)
Supplement: Supplementary file 1 — Supporting Information [file MCO2-6-e70068-s001.docx]

**Targeting intracellular cancer proteins with tumor-microenvironment-responsive bispecific nanobody-PROTACs for enhanced therapeutic efficacy**

**Running title:** **Novel protein degraders for cancer treatment**

Changping Deng^1,2^, Jiacheng Ma^4^, Yuping Liu^3^, Xikui Tong^1^, Lei Wang^1^, Jiayi Dong^1^, Ping Shi^1^, Meiyan Wang^5^, Wenyun Zheng^3*^, Xingyuan Ma^1*^

^1^State Key Laboratory of Bioreactor Engineering, East China University of Science and Technology, Shanghai 200237, P. R. China

^2^Key Laboratory of Systems Biomedicine (Ministry of Education), Shanghai Center for Systems Biomedicine, Shanghai Jiao Tong University, Shanghai 200240, P. R. China

^3^Shanghai Key Laboratory of New Drug Design, School of Pharmacy, East China University of Science and Technology, Shanghai 200237, P. R. China

^4^The Department of Information Engineering, The Chinese University of Hong Kong, Hong Kong 999077, P. R. China

^5^School of Medicine, Shanghai University, Shanghai 200444, P. R. China

* To whom correspondence should be addressed.

Prof. Dr. Wenyun Zheng, Email: zwy@ecust.edu.cn

Prof. Dr. Xingyuan Ma, E-mail: [maxy@ecust.edu.cn](mailto:maxy@ecust.edu.cn)

Full postal: Laboratory of Biopharmaceutical and Cell Engineering, School of Biological, Engineering State Key Laboratory of Bioreactor Engineering, East China University of Science and Technology, 130 Meilong Road, P.O. Box No. 365, Shanghai, 200237, P. R. China

**SUPPLEMENTARY INFORMATION:**

**Table S1:** Information on RL, RVL, R14L, KVN and K14N proteins was recorded.

**Figure S1:** Molecular docking of nanobodies to antigens.

**Figure S2:** Schematic diagram of vector construction in this study.

**Figure S3:** UMUC-3-EGFP cells were treated with MG132, Baf, RVL, or R14L for 12 h and then photographed by fluorescence microscopy. Scale bar: 100 μm.

**Figure S4:** The HPA database analysis of PD-L1, Furin, and Survivin proteins expression levels in bladder tissue. Scale bar: 200 μm.

**Figure S5:** MTT assay analysis of KVN and K14N proteins. A: MTT analysis of 5637 cells treated with KVN and K14N proteins at 24 h, 48 h, and 72 h. B: MTT analysis of UMUC-3 cells treated with KVN and K14N proteins at 24 h, 48 h, and 72 h. C: MTT analysis of A375 cells treated with KVN and K14N proteins at 24 h, 48 h, and 72 h.

**Figure S6:** The half degradation concentration (DC_50_) of KVN and K14N were determined by western-blot.

**Figure S7:** All original western-blot images in this study.

**METHODS**

**Bioinformatics analysis**

Analysis of the mRNA and protein expression levels of *PD-L1,* *Furin*, and *Survivin* genes in normal and cancerous tissues using the online databases. TCGA website is “http://ualcan.path.uab.edu/analysis.html”, and The Human Protein Atlas (THPA) website is “https://www.proteinatlas.org”. Meanwhile, “https://gramm.compbio.ku.edu/request” and “https://zdock.umassmed.edu/” websites are chosen to verify the binding of nanobodies to antigens.

**Vector construction and protein expression**

A total of six vectors were constructed, and six proteins were expressed in this study, and their components were “-RRRRRRRRR- (R_8_)”, “-GSG-”, “-RVRR-”, “VHLL”, “14aa”, “LaG16”, “EGFP”, “KN035”, and “Nb4A”. “LaG16” and “KN035” were synthesized by Sangon Biotech (Shanghai) Co., Ltd. The vectors constructed include R_8_-LaG16 (named RL), R_8_-VHLL-GS-LaG16 (named RVL), R_8_-(GSG)_2_-RVRR-14aa-LaG16 (named R14L), KN035-GSG-RVRR-EGFP (named KE), KN035-GSG-RVRR-VHLL-Nb4A (named KVN), and KN035-GSG-RVRR-14aa-Nb4A (named K14N). These fragments had *Nde* I and *Xho* I sites at both ends, and the vectors chosen were also all pET-22b. The fragments were ligated to the vector by homologous recombinant enzymes according to the manufacturing instructions, respectively. The constructed vectors were successfully transferred to *Trans*B(DE3) strains (the strain contains mutated thioredoxin reductase and glutathione reductase, TransGen Biotech). The expression conditions for RL, RVL, and R14L were 18 ℃, 0.5 mM isopropyl-beta-D-thiogalactopyranoside (IPTG), and induction for 16 h. The target proteins were existed in supernatant of the bacteria lysate. Also, the expression conditions for KE, KVN, and K14N were 30 ℃, 0.75 mM IPTG, and induction for 14 h. The target proteins were located in the supernatant of the bacteria of broken. The supernatant proteins were purified using the His‐tagged protein purification kit (Shanghai Sangon Co., Ltd). All information of proteins can be found in the **Supplementary Data Table S1**.

**Western-blot**

The resulting cells were collected and lysed with RIPA buffer (high) (Solarbio) containing 1 mM phenylmethylsulfonyl fluoride (PMSF, Solarbio), and the supernatant was collected by centrifugation. After quantification by the BCA kit, the sample size was set at 30 μg. After a series of operations such as SDS-PAGE, membrane transfer, and antibody incubation, the samples were developed with ECL solution and analyzed by ImageJ software for grey scale values.

**EGFP fluorescence intensity assay**

Three methods were chosen to determine EGFP fluorescence intensity in this study. The first was to directly observe the fluorescence expression level using the fluorescence inverted microscopy (Olympus, Japan) by adding each of the three proteins RL, RVL, and R14L to the same density of UMUC-3-EGFP cells at the same concentration, respectively, incubating for 48 h and then photographing. EGFP channel (λex 488 nm and λem 525 nm). The second method was to test the fluorescence intensity in a 96-well plate using the fluorescent microplate reader in the same way as described above. Fluorescent channel (λex 485 nm and λem 528 nm). The third method was to detect the fluorescence intensity using flow cytometry. The incubated cells were washed three times with phosphate-buffered saline (PBS) buffer, then digesting the treated cells with trypsin, washing the digested cells with PBS buffer, and finally resuspending them with 100 μL of PBS buffer and mixing them with gentle blowing. The cells were then assayed by flow cytometry (Beckman Coulter, USA). For each experiment, 1 × 10^4^ cells were counted.

**Protein internalisation assay**

The expression of purified KE proteins was used to validate the internalization of the proteins. The 5637, UMUC-3, and A375 cells were selected for analysis. KE protein was added to 5637, UMUC-3, and A375 cells at the same concentration and incubated for 2 h, 4 h, 6 h, and 8 h, then washed three times with PBS buffer and fixed in 4% paraformaldehyde for 15 min, then washed three times with PBS buffer and 1 × 4',6-diamidino-2-phenylindole (DAPI) was added and left to stand for 10 min away from light, then washed 3 times with PBS buffer for confocal photography. Tracing the internalization of KE proteins in cells by EGFP expression. FITC channel (λex 488 nm and λem 525 nm), and DAPI channel (λex 364 nm and λem 454 nm).

**Characterization of the working proteins**

The working protein contained KVN and K14N. The homology modeling of KVN and K14N was first carried out through the online website SWISS-MODEL (https://swissmodel.expasy.org/interactive) to obtain predicted-tertiary structures to determine the spatial distribution states of each component of the working proteins. Meanwhile, the secondary structure of the protein was assessed using circular dichroism (CD) spectrometer. Two proteins, KVN (0.3 mg/mL in PBS buffer) and K14N (0.3 mg/mL in PBS buffer), were placed at 25 °C, 37 °C and 45 °C for 24 h before recording the CD spectra of the samples from 260 to 200 nm in a 1 mm quartz cell.

**Furin protease cleavage analysis *in vitro***

Furin protease was incubated with 2 µg KVN or K14N in 20 mM HEPES, 0.1% Triton X-100, 1 mM CaCl_2_, 0.2 mM β-mercaptoenthanol (pH 7.4, 37 °C) in a 25 µl reaction. Moreover, the reaction times were 10 min, 30 min, 1 h, 2 h, 4 h, and 6 h, respectively. SDS-PAGE visualizes the separation of reaction products.

**MTT assay**

MTT assay was used to determine the cytotoxicity of KVN and K14N (Cell viability and IC_50_). 5637 cells, UMUC-3 cells, and A375 cells (2 × 10^4^ cells/well) were seeded into a 96-well plate and cultured overnight before incubating with KVN or K14N (0, 30, 60, 90, 120, and 150 μg/mL) for 24 h, 48 h, and 72 h, respectively. After that, 20 μL of MTT solution (5 mg/mL) was added to each well and incubated for another 4 h in the incubator. The produced formazan was dissolved in dimethyl sulfoxide (DMSO, 150 μL), and the absorbance was detected at 490 nm by the microplate reader (Biotek Instruments Inc., United States). Analyze the data by GraphPad Prism software to determine its cell viability and IC_50_ value.

**Cell apoptosis assay**

The 5637, UMUC-3, and A375 cells were inoculated into 6-well plates at 5 × 10^5^ per well, respectively. Moreover, these cells were treated with KVN (85 μg/mL) or K14N (85 μg/mL) for 48 h. Then the apoptosis percentage of the above-treated cells was measured using an Annexin V-FITC/PI apoptosis kit and flow cytometry (Beckman Coulter, USA).

**Cloning formation assay**

The 5637, UMUC-3, and A375 cells were seeded into a 6-well plate at 2 × 10^3^ per well, respectively, and treated with KVN (85 μg/mL) or K14N (85 μg/mL) for 48 h. Subsequently, the medium was changed every two days, and after 14 days of incubation, the cells were washed three times with PBS buffer and fixed with 4% paraformaldehyde solution for 15 min. The cells were then washed three more times with PBS buffer, and finally, all cells were stained with 0.1% crystalline violet solution and imaged.

**Cell migration assay**

Wound healing assays determined the migration of cells. Briefly, 5637, UMUC-3, and A375 cells were seeded at 2 × 10^5^ per well in 12-well plates and grown to 90% confluence. Immediately after, KVN (85 μg/mL) or K14N (85 μg/mL) was added and treated for 48 h. Replace the medium with medium containing 1% fetal bovine serum after scratching was complete. A sterile pipette tip was utilized to create artificial gaps in the cellular monolayer. These wounds were then demarcated and meticulously documented using a high-resolution digital camera system. Subsequently, the cell migration distances were meticulously quantified employing the HMIAS-2000 software program, which offered a precise analysis of the cellular movement.

**Cell viability assays**

The 5637, UMUC-3, and A375 cells were respectively plated onto 12 wells at a density of 5.0 × 10^4^ per well at 37 ℃ in a 5% CO_2_ incubator. After 24 h, treated with KVN (85 μg/mL) or K14N (85 μg/mL) for 48 h, respectively. After removing the growth medium, the cells were washed with cold PBS buffer. We then used the Calcein/PI assay kit to assess cell viability and cytotoxicity. The cells were incubated with Hoechst 33342 for 10 min to stain the nuclei, followed by two more washes with PBS. Finally, the cells were imaged under a fluorescent microscope using specific channels for FITC, PI, and Hoechst 33342 to visualize cell viability, cytotoxicity, and nuclear morphology.

**Indirect immunofluorescence assay**

The 5637 cells was plated onto the glass bottom cell culture dish (20 mm diameter, biosharp) at a density of 2.0 × 10^4^ cells/well at 37 ℃ in a 5% CO_2_ incubator. After 24 h, the working proteins KVN or K14N were added to the dish at a certain concentration and the cells were treated at 12 h, 24 h, and 48 h, respectively. The treated cells were gently washed 3 times with pre-cooled PBS buffer, then fixed with 4% paraformaldehyde for 15 min, and then gently washed 3 times with pre-cooled PBS buffer. The cells were then treated with permeabilizing solution (PBS solution containing 0.2% Triton X-100) for 5 min and washed gently with pre-cooled PBS buffer three more times. Cells were then blocked with blocking solution (PBS solution containing 0.1% Tween-20, 1% Bovine Serum Albumin (BSA), and 22.52 g/L glycine) and incubated at room temperature on a horizontal rotary shaker at low speed for 1 h. Immediately afterward, samples were washed three times with PBST solution (PBS solution containing 0.1% Tween-20), followed by the primary antibody was then diluted in antibody diluent (PBS solution containing 0.1% Tween-20 and 1% BSA) at the official recommended rate, and incubated on a horizontal rotating shaker at room temperature for 1 h. After incubation of the primary antibody, the sample was washed three times with PBST. Similarly, the fluorescently labeled secondary antibody was diluted in antibody diluent at the officially recommended rate and incubated for 1 h at room temperature on a horizontal rotating shaker at low speed, protected from light, after which the samples were washed 3 times with PBST. Finally, the samples were incubated for 10 min with 1 × DAPI solution for nucleus staining. After washing the samples 3 times with PBST, they were prepared for confocal microscopy.

**3D tumor spheroid growth inhibition**

The 5637 cell line and UMUC-3 cell line were selected for the construction of 3D tumor spheroids. The initial cell numbers were 5 × 10^4^, and 5637 cells formed tight spheres after 24 h of culture, while UMUC-3 cells could form tight spheres only after 96 h of culture. Then, the corresponding concentration of working proteins was added to the culture medium, and the medium was changed every two days. The intelligent living cell imaging system (Gnano, Shanghai, China) was chosen to observe the status of 3D tumor spheroids at all times, and photographs were taken for recording.

***In vivo* antitumor efficiency**

Five-week-old female BALB/c nude mice were randomly divided into PBS, KVN, and K14N groups (n = 5 for each group) (Nanjing Junke Biological Co., Ltd., China). Since the 5637 cell line was difficult to tumorize, the UMUC-3 cell line was selected for nude mouse tumorigenesis experiments. For the cell-derived xenograft (CDX) model, 1.0 × 10^7^ cells in 100 μL DMEM (non-resistant, serum-free, phenol red-free) were injected at the right side of the back of each mouse, and the tumor size was measured every 3 days following the formula volume (mm^3^) = (length) × (width)^2^/2. Nude mice in the experimental group were injected with KVN and K14N proteins at a concentration of 5 mg/kg body weight every three days. Also, the weight of the mouse was measured every 3 days. All mice were euthanized 15 days after treatment, tumor sections were embedded, hematoxylin and eosin (HE) staining, and immunohistochemistry (IHC) staining. Furthermore, the major organs of nude mice also required HE staining (Shanghai RIBIOLOGY Technology Co., Ltd., China).

**Table S1**

| **Name** | **Theoretical size** | **Theoretical pI** | **Homology modeling** |
| --- | --- | --- | --- |
| RL | 17.07 kDa | 10.31 | 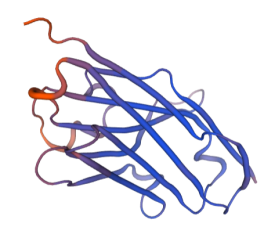 |
| RVL | 17.95 kDa | 10.18 | 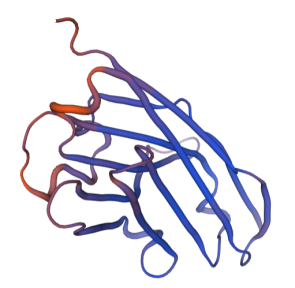 |
| R14L | 19.54 kDa | 11.12 | 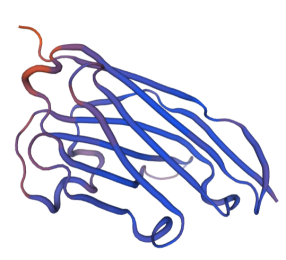 |
| KVN | 29.81 kDa | 9.30 | 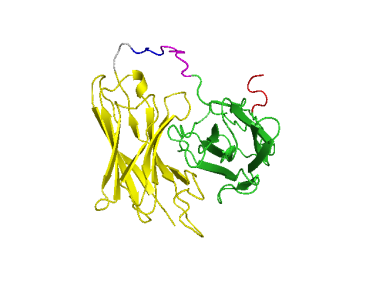 |
| K14N | 30.58 kDa | 9.63 | 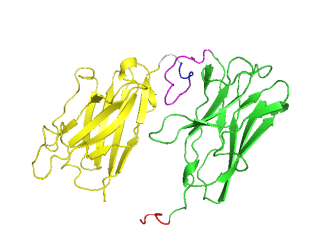 |


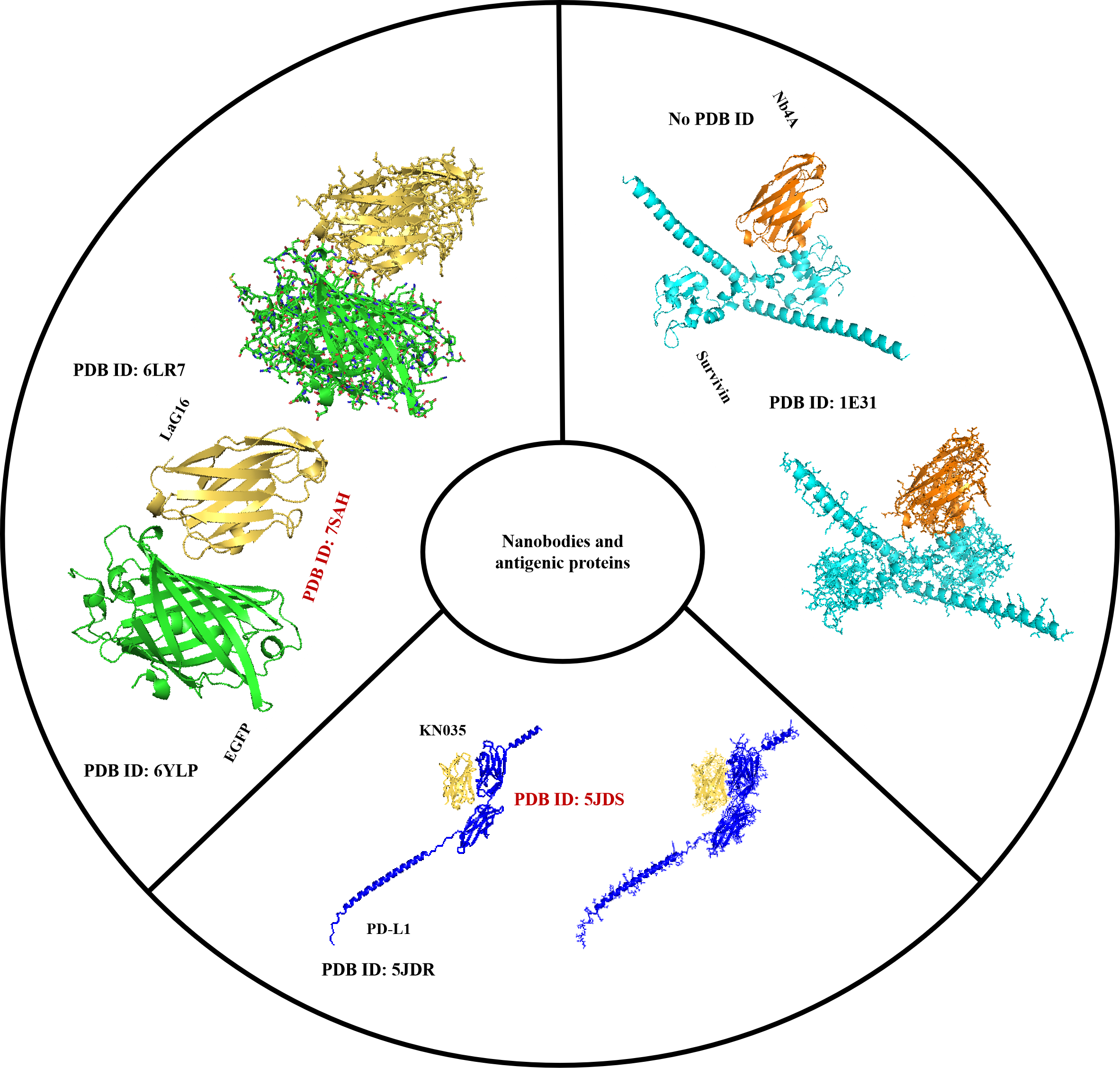


**Figure S1**


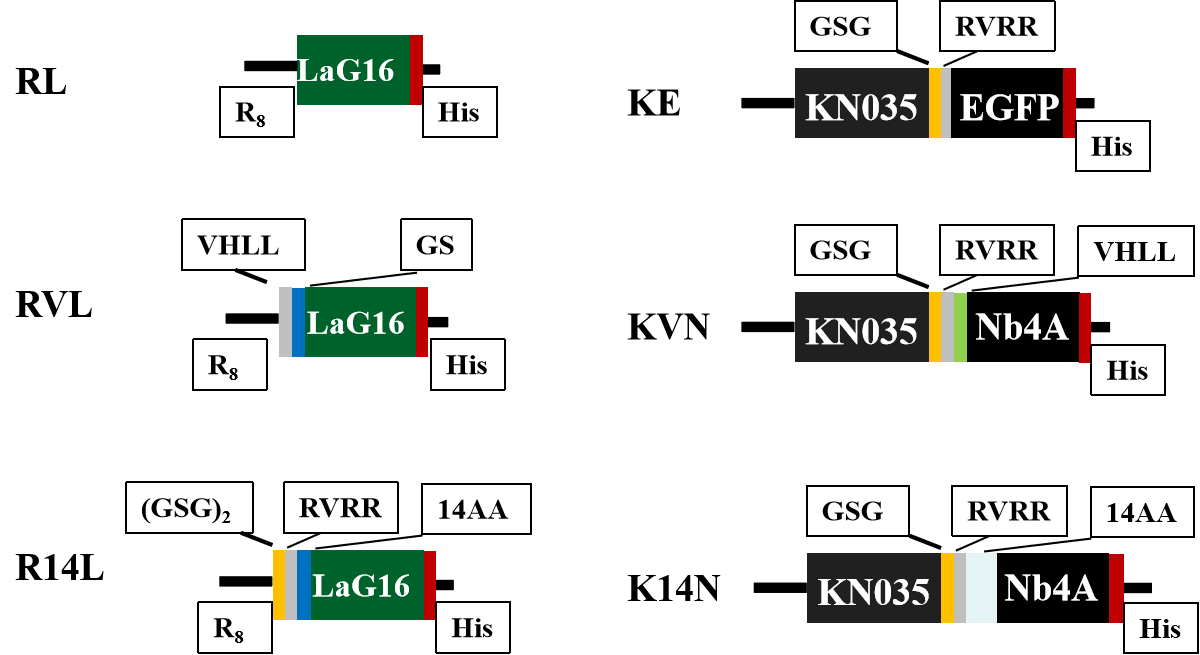


**Figure S2**


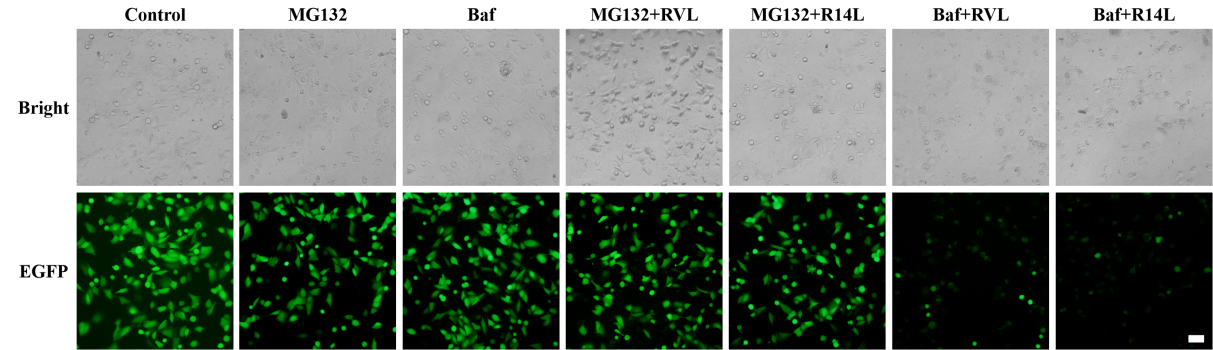


**Figure S3**


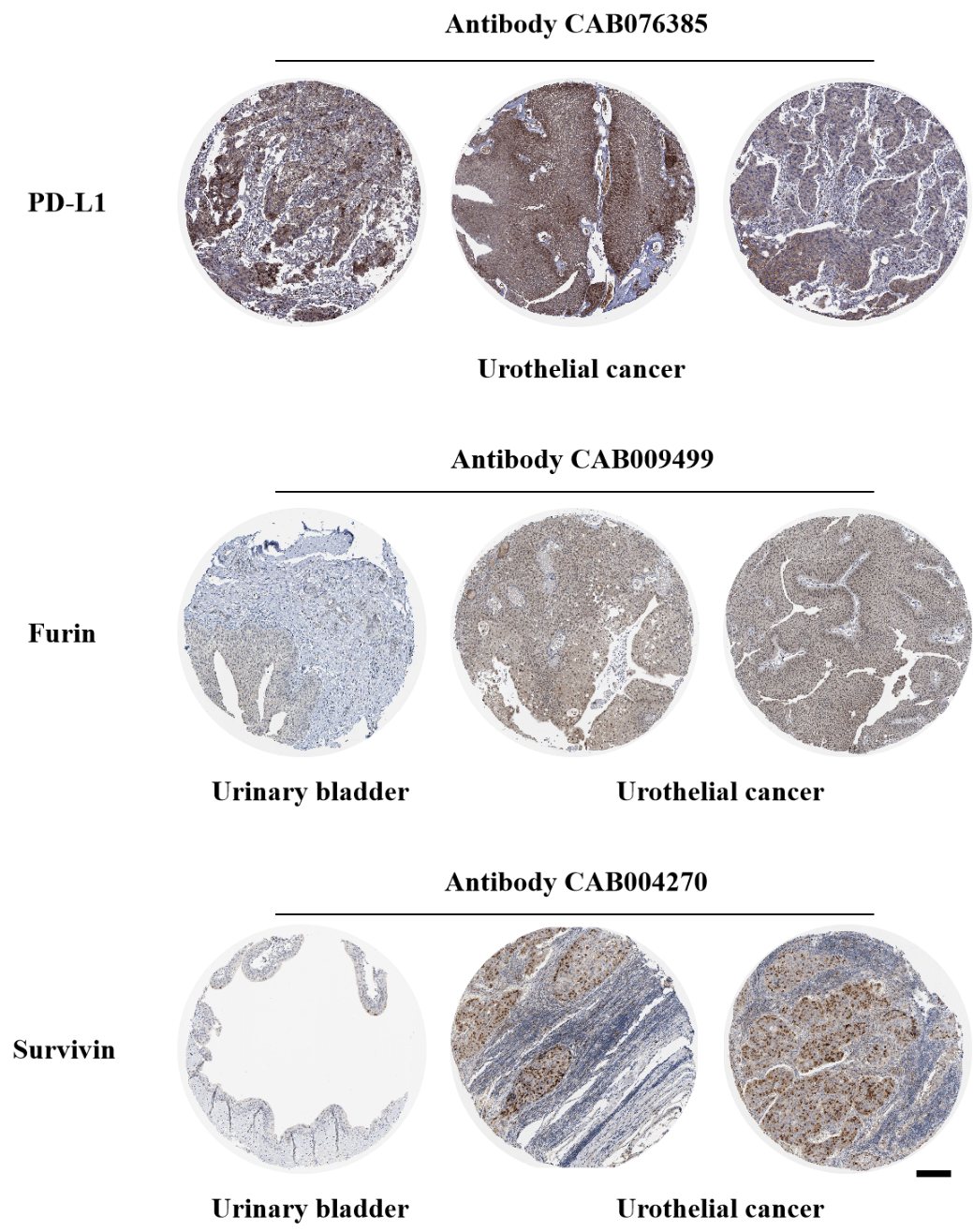


**Figure S4**


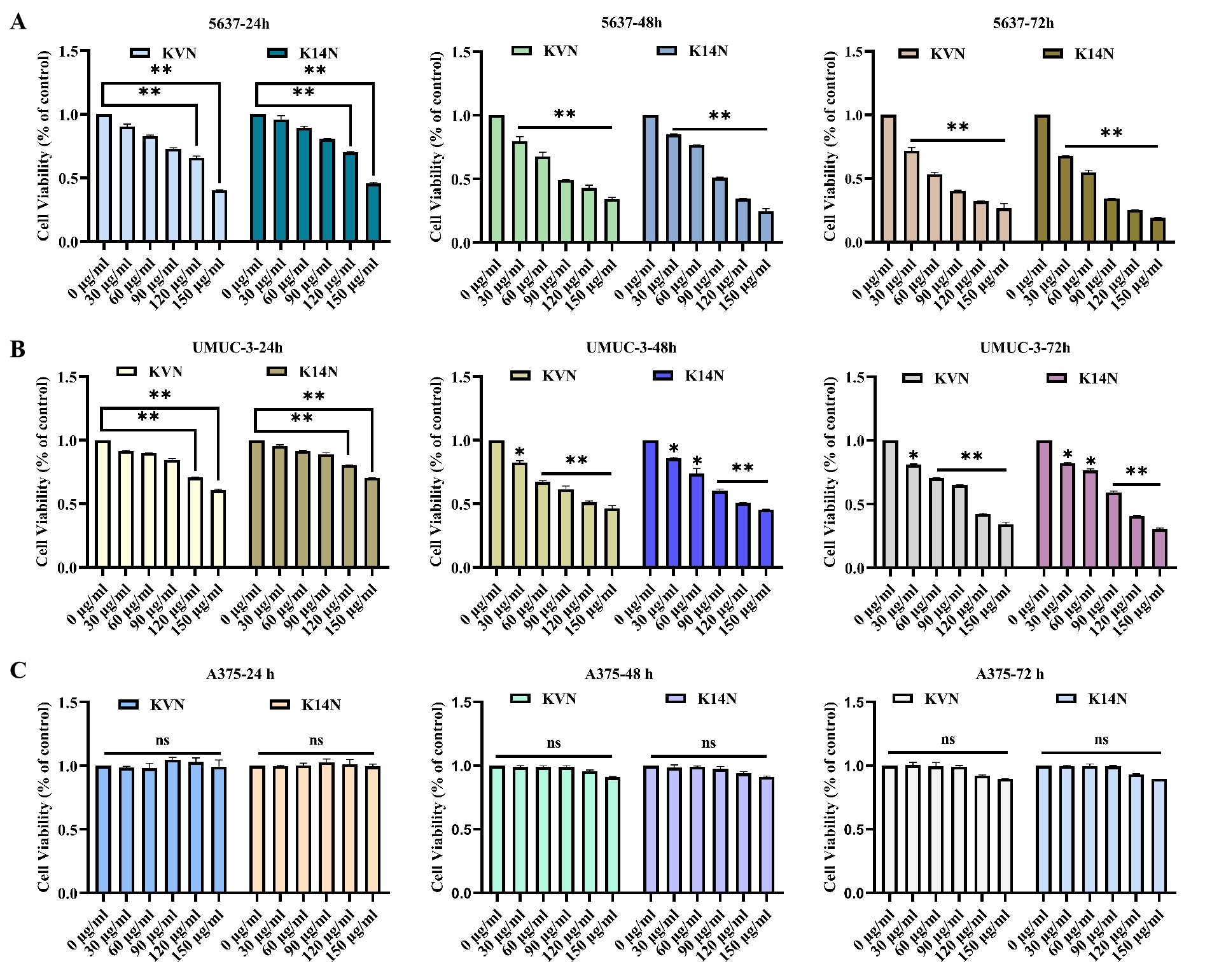


**Figure S5**


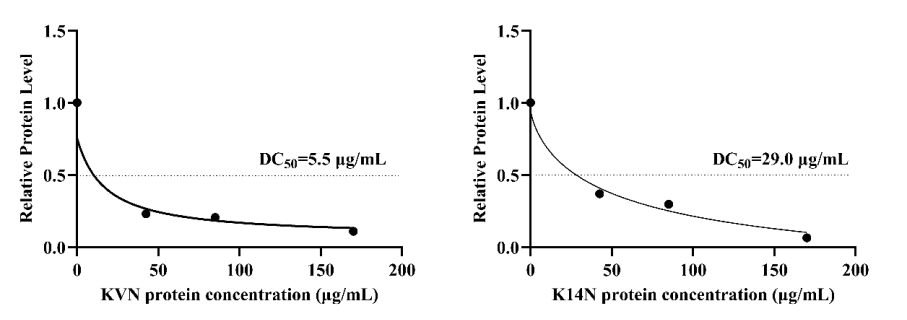


**Figure S6**

**
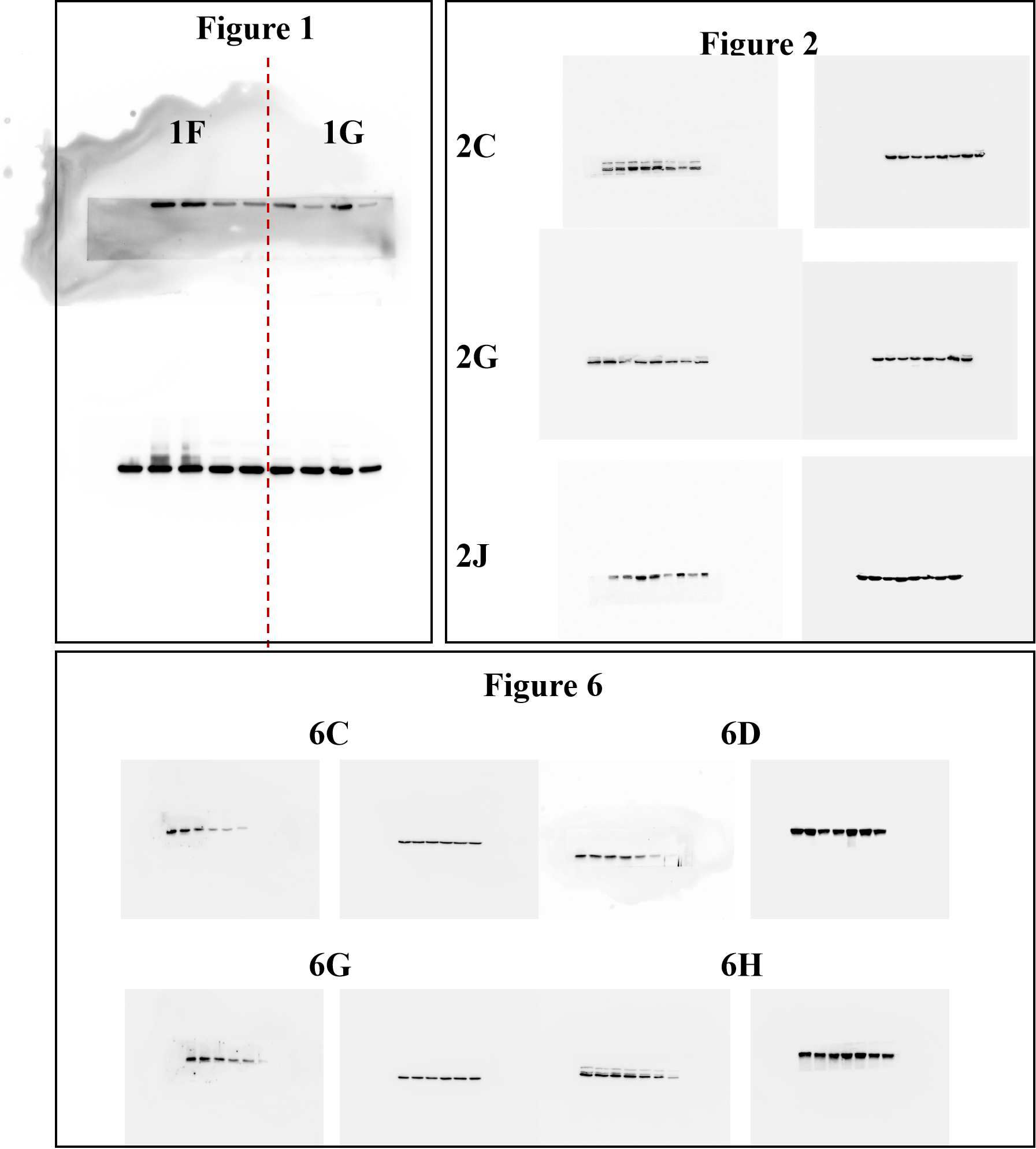
**

**Figure S7**
